# A polysaccharide deacetylase from *Puccinia striiformis* f. sp*. tritici* is an important pathogenicity gene that suppresses plant immunity

**DOI:** 10.1111/pbi.13345

**Published:** 2020-03-04

**Authors:** Qiang Xu, Jianfeng Wang, Jinren Zhao, Jinghua Xu, Shutian Sun, Huifei Zhang, JiaJie Wu, Chunlei Tang, Zhensheng Kang, Xiaojie Wang

**Affiliations:** ^1^ State Key Laboratory of Crop Stress Biology for Arid Areas and College of Plant Protection Northwest A&F University Yangling Shaanxi China; ^2^ State Key Laboratory of Crop Biology Shandong Agricultural University Tai’an Shandong China

**Keywords:** *Puccinia striiformis* f. sp. *Tritici(tritic)*, polysaccharide deacetylase, PAMP‐triggered immunity (PTI), wheat (*Triticumaestivum, Triticum aestivum*)

## Abstract

The cell wall of filamentous fungi, comprised of chitin, polysaccharide and glycoproteins, maintains the integrity of hyphae and protect them from defence responses by potential host plants. Here, we report that one polysaccharide deacetylase of *Puccinia striiformis* f. sp. *tritici* (*Pst*), Pst_13661, suppresses Bax‐induced cell death in plants and Pst_13661 is highly induced during early stages of the interaction between wheat and *Pst*. Importantly, the transgenic wheat expressing the RNA interference (RNAi) construct of *Pst_13661* exhibits high resistance to major *Pst* epidemic races CYR31, CYR32 and CYR33 by inhibiting growth and development of *Pst*, indicating that Pst_13661 is an available pathogenicity factor and is a potential target for generating broad‐spectrum resistance breeding material of wheat. It forms a homo‐polymer and has high affinity for chitin and germ tubes of *Pst* compared with the control. Besides, Pst_13661 suppresses chitin‐induced plant defence in plants. Hence, we infer that Pst_13661 may modify the fungal cell wall to prevent recognition by apoplastic surveillance systems in plants. This study opens new approaches for developing durable disease‐resistant germplasm by disturbing the growth and development of fungi and develops novel strategies to control crop diseases.

## Introduction

Frequent natural disasters and changing climate threaten global agricultural production. Chronic diseases caused by pathogens, such as filamentous fungi, oomycetes, bacteria and pests present urgent issues that must be addressed immediately (Fang and Ramasamy, 2015). For example, *Phytophthora infestans* causes potato late blight and rust fungi, especially wheat rusts (*Puccinia striiformis* f. sp. *tritici*, *Puccinia*. *graminis* f. sp. *tritici* and *Puccinia triticina*) threaten the production of economically important crops. Evolution and migration of pathogens are occurring at increasing rates due to inappropriate expansion of planting resistant plants in crop‐producing regions, resulting in unexpected reductions in grain yield (Singh *et al.*, 2011). The sudden emergence of Ug99 strain, which was able to infect previously resistant wheat varieties, threatened wheat production and grain security throughout many regions of the world (Singh *et al.*, 2011). Thus, a thorough knowledge of pathogenic mechanism of pathogens would be a beneficial measure to minimize disease prevalence and create resistant materials.

The infection structures of filamentous biotrophic pathogens absorb nutrients from host cells to survive in living plant tissue. These structures, for example, haustoria, are usually tightly appressed to the outside of host cells and are exposed to hostile plant apoplast. Therefore, inhibiting or escaping plant immune responses and maintaining the integrity and development of the hyphae are prerequisites for survival of the pathogen (Asai and Shirasu, 2015; Thines and Kamoun, 2010). The cell wall of filamentous fungi, comprised of chitin, glucan and proteins, provides cellular structure and hyphal integrity (Feofilova, 2010). Chitin, a polymer of N‐acetyl‐D‐glucosamine, is main component of the fungal cell wall and can be cross‐linked with polysaccharides to protect the cell wall from hydrolysis and maintain the integrity of hyphae (Feofilova, 2010; Kombrink *et al.*, 2011; Goldman & Vicencio, 2012). Chitin is regarded as one of most abundant polysaccharides and is a component of fungal cell walls but not plant cell walls (Zhao *et al.*, 2010). Multilayers of plant immunity mechanisms are challenged by the corresponding pathogens through natural coevolution. The components of the fungal cell wall are conserved structures that serve as perfect targets for the recognition of plant immune responses. For example, bacterial flagellin and chitin oligomers as pathogen‐associated molecular patterns (PAMPs) are recognized by pattern recognition receptors (PRRs) on the plant cell membrane and stimulate the plant’s basal defence response, such as the accumulation of reactive oxygen species (ROS), the amplification of MAPK cascade and the increasing callose precipitate on the plant cell wall (Jones & Dangl, 2006; Nicaise *et al.*, 2009; Iwasaki & Medzhitov, 2010). In addition, plants secrete antibacterial metabolites (phenols and alkaloids) and enzymes (hydrolases, chitinases and proteases) to suppress invading pathogens (Deising *et al.*, 1995; Piasecka *et al.*, 2015). Plant chitinases are highly induced as one type of degrading enzyme against biotrophic fungi during pathogen invasion (Brogue *et al.*, 1991). The chitin layers are degraded by chitinases from plant cells and form small chitin fragments, designated chito‐oligosaccharides. The weakened cell walls of filamentous fungi are more accessible to the attack of hydrolases, which ultimately leads to the accelerated death. More directly, the freed chito‐oligosaccharides released from the cell walls of fungal pathogens are more easily perceived as an elicitor to stimulate basal immunity against pathogen invasion (Hamel and Beaudoin, 2010). A range of responses can severely discourage further development of pathogens in plant tissues. These basal defence responses of plants are collectively called PAMP‐triggered immunity (PTI). However, cell wall‐modifying enzymes of fungi successfully mask the recognition sites of hyphae in the interaction between pathogens and host. The secreted LysM protein 1 (Slp1) is a good example for competitively binding the chitin oligosaccharides against the chitin elicitor binding protein (CEBiP) on the plant cell membrane (Mentlak *et al.*, 2012). Effector Avr4 from *Cladosporiumfulvum* binds chitin in cell walls to protect this fungus against plant chitinases (Van den Burg *et al.*, 2006). The self‐protective mechanisms of fungal pathogens responding to plant attack are effective measures. These polysaccharide/chitin deacetylases seem like counter defensive virulence factors to protect the cell wall of pathogens from degradation and recognition by host surveillance systems. Due to the highly conserved structure in the evolution of PAMPs and conserved pathogen factors, knowledge of these conserved factors may provide a good opportunity for developing broad‐spectrum resistance to various pathogens (Boyd *et al.*, 2013).

Wheat stripe rust caused by *Puccinia striiformis* f. sp. *tritici* (*Pst*) threatens the global crop production and food security. Due to obligate parasitism of *Pst* and the complex hexaploid genome of wheat, little is known about the physiological metabolism and virulence factors that influence virulence and the growth and development of *Pst*. The whole‐genome sequencing of *Pst* provided effective bioinformatic approaches to identify virulence factors. Based on the genomic analysis of *Pst* CYR32, we obtained some cell wall‐hydrolysing enzyme (CWHE) genes and other carbohydrate‐degrading enzymes (Zheng *et al.*, 2013). Approximately 20 members, including gene pairs cluster of a polysaccharide deacetylase family, were extracted and analysed. We chose one cluster, including Pst_13661 and Pst_13662, with high similarity in amino acid sequence for further study. *Pst_13661*, which is highly expressed in *Pst*–wheat interaction, was selected to investigate its function. Interestingly, Pst_13661 formed homo‐polymers that accumulated in the plant apoplast and suppressed Bax‐induced cell death. As a polysaccharide deacetylase, Pst_13661 has high affinity to chitin and fungal hyphae *invitro* and it also suppresses chitin‐induced plant defence. The transgenic wheat of silencing *Pst_13661* exhibits high resistance to *Pst* by increasing the accumulation of ROS and inhibiting growth and development of *Pst.* We speculate that Pst_13661 may have positive roles in modifying fungal chitin to avoid recognition. Taken together, this study encourages us to think about how to use PAMPs and other inherent factors to inhibit the growth and development of pathogens. This study will open new avenues for the creation of durable disease‐resistant breeding material and novel strategies to control crop diseases.

## Results

### Pst_13661 from *Pst* belongs to the polysaccharide deacetylase family

**Figure 1:**
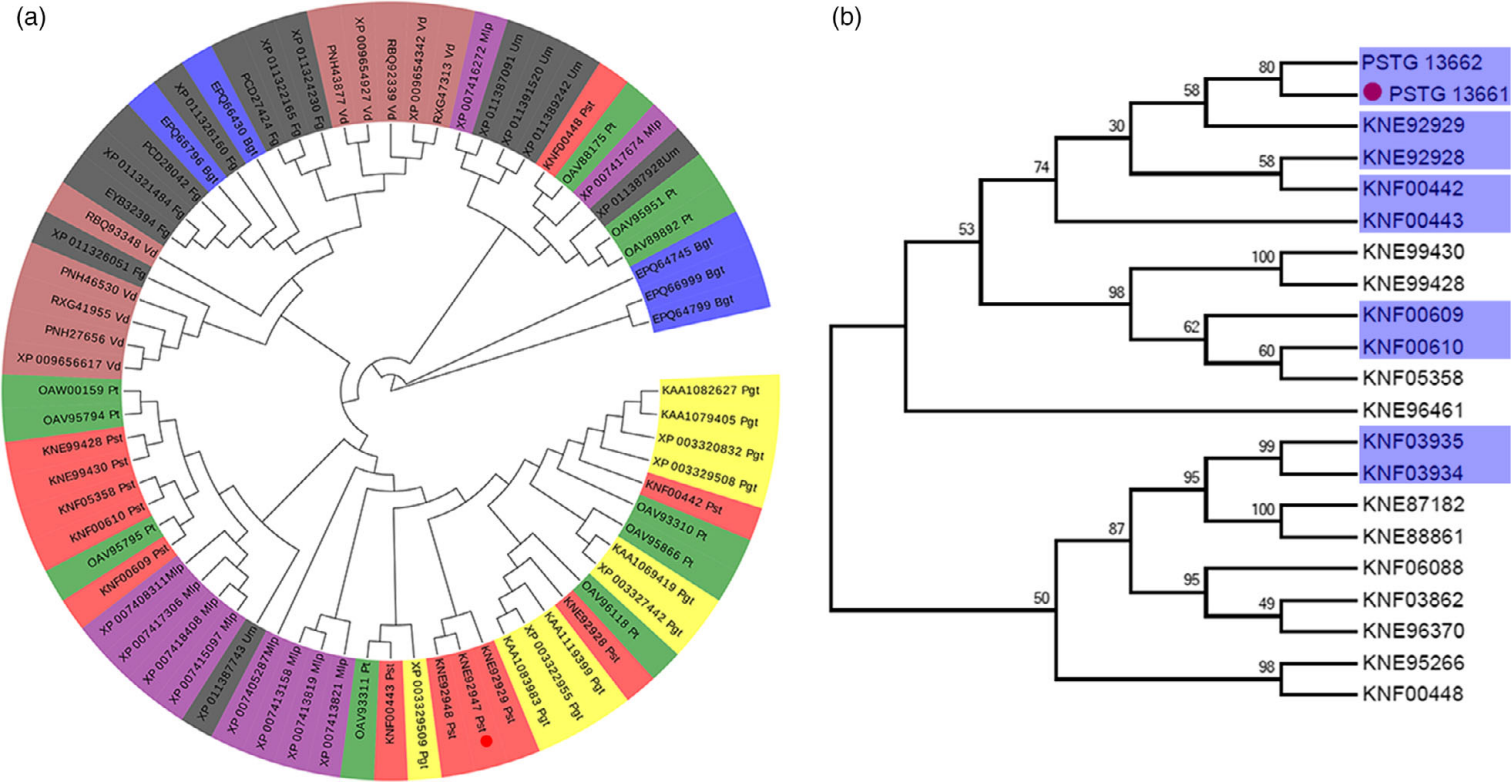
Phylogenetic analysis of the polysaccharide deacetylase family in biotrophic fungi. (a) Phylogenetic analysis of polysaccharide deacetylases from biotrophic fungi using MEGA5. Branches are labelled with GenBank accession numbers and the organisms. The red circle shows *Pst_13661*. *Pucciniastriiformis* f. sp. *tritici* (*Pst*), *Puccinia triticina* (*Pt*), *Puccinia graminis* f. sp. *tritici* (*Pgt*), *Blumeria graminis* f. sp. *tritici* (*Bgt*), *Melampsora larici‐populina* (*Mlp*), *Fusarium graminearum* (*Fg*), *Ustilago maydis* (*Um*) and *Verticillium dahlia* (*Vd*) were retrieved from National Center for Biotechnology Information (NCBI) database. (b) Phylogenetic analysis of the polysaccharide deacetylases family of *Pst* using MEGA5. The gene sequences were obtained from the genome of *Puccinia striiformis* f. sp. *tritici PST‐78* in the website (http://fungi.ensembl.org/). The blue shadow indicates the gene clusters, and the red circle shows the gene *Pst_13661*.

Based on genome and secretome analyses of *Pst* CYR32, there are several categories of cell wall‐hydrolysing enzyme (CWHE) genes and carbohydrate‐active enzymes (CAZY) that are conserved genes in biotrophic fungi (Zheng *et al.*, 2013). Due to the conservation and significance of polysaccharide deacetylases (PDAs) in biotrophic fungi, phylogenetic analysis indicated that, compared with *Blumeria graminis* f. sp. *tritici*, *Magnaporthe oryzae* and *Ustilago maydis*, the PDAs of *Pst* aggregated into groups with high similarity to other rust fungi (Figure 1a). Interestingly, we found that five gene pairs cluster (10 members) of the polysaccharide deacetylase family were arranged in genomic clusters in *Pst* (Figure 1b). More interestingly, the similarity in their amino acid sequences was achieved 47%–82% and seven members have a signal peptide and three have transmembrane regions (Table S1). *Pst_13661* and *Pst_13662* were two members with typical 24‐aa signal peptides and a conserved polysaccharide deacetylase domain at the N‐terminus. In addition, *Pst_13661* and *Pst_13662* were in one cluster of PDAs in the *Pst* genome (Figure S1), and their nucleotide sequences shared more than 80% similarity and the amino acid sequence was 82% similar (Figure S2; Table S1). Thus, PDAs are conserved in filamentous fungi, especially in rust fungi.

### *Pst_13661* is induced during the early stages of infection

To determine the transcription expression level of *Pst_13661* and *Pst_13662* during the wheat–*Pst* interaction, we evaluated their transcript levels from urediniospores, germ tubes and several important infection stages. As shown in Figure S3, *Pst_13661* was highly induced in the early infection stages, including at 12 and 48 hpi, about 2.5, twofold changes compared with the control urediniospores, respectively. Then, the transcripts levels declined from peak to a low level at 72–96 hpi. However, the transcript level of *Pst_13662* was low at all infection stages compared with urediniospores (Figure S3). The results suggest that Pst_13661 is involved in *Pst*–wheat interaction.

### Pst_13661 suppresses Bax‐induced cell death in *Nicotiana benthamiana*

**Figure 2:**
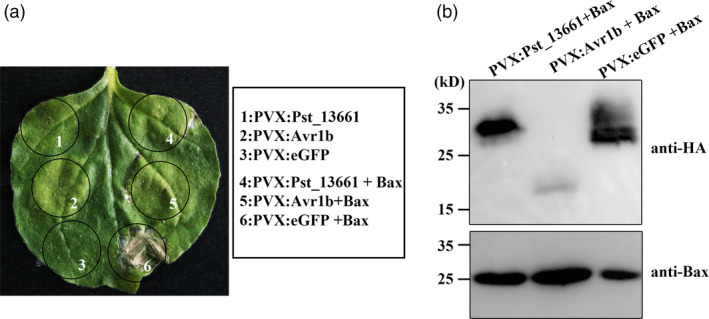
*Pst_13661* suppresses Bax‐induced cell death in *Nicotiana benthamiana*. (a) Transiently expressing *Pst_13661* 24 h prior to infiltrating with *Agrobacterium* strain carrying Bax in *Nicotiana benthamiana*. eGFP was a negative control, and Avr1b was a positive control. Images were photographed 7 days after inoculation with *Agrobacterium* carrying the corresponding vectors. (b) Total proteins from *Nicotiana benthamiana* were extracted at 72 h after infiltration with *Agrobacterium,* and proteins Pst_13661:HA (28 kD), Avr1b:HA (15 kD) and eGFP:HA (25 kD) were detected by Western blot with anti‐HA antibody. Protein Bax (22 kD) was detected with anti‐Bax antibody.

The inhibition assay of the cell death induced by the mouse protein Bax is a screening criterion for virulence factors. Therefore, we tried to detect the virulence function of Pst_13661 in *N*. *benthamiana*. The negative control eGFP did not suppress cell death (Figure 2a). However, like the positive control, effector Avr1b from oomycetes, Pst_13661, suppressed Bax‐induced cell death in *N*. *benthamiana* (Figure 2a). In addition, the Western blot assay confirmed that Pst_13661 and Bax proteins were correctly expressed in *N*. *benthamiana *(Figure 2a). Western blot analysis with anti‐HA and anti‐Bax antibodies confirmed the expression of eGFP (25 kD), Pst_13661 (28 kD), Avr1b (15 kD) and Bax (22 kD), respectively, *in vivo* (Figure 2b).

### Silencing *Pst_13661* impairs the pathogenicity of *Pst*

**Figure 3:**
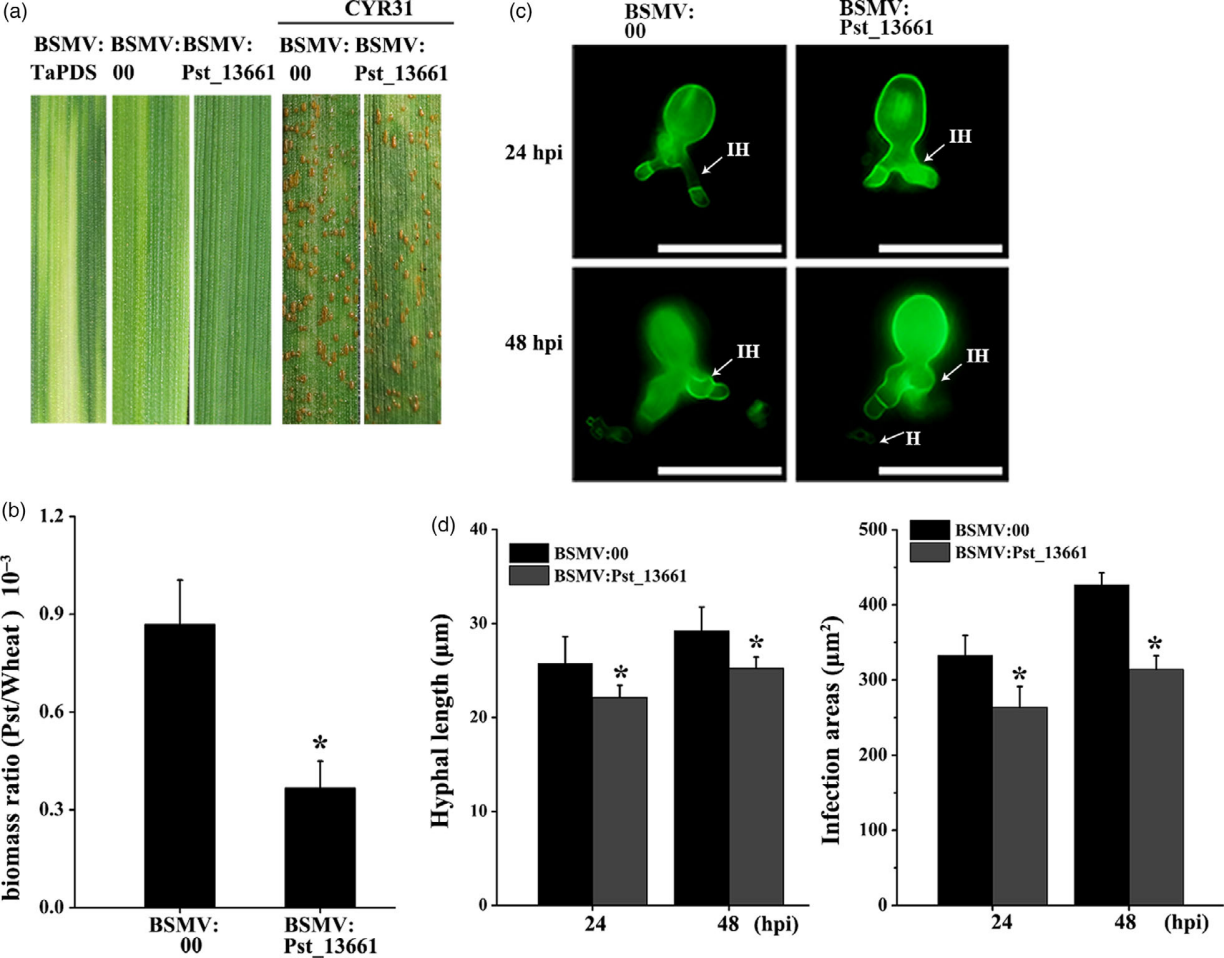
BSMV‐mediated silencing *Pst_13661* in wheat. (a) Mild chlorotic mosaic symptoms and disease phenotype on the seedling leaves inoculated with BSMV:TaPDS, BSMV:00 (empty vector) and BSMV:Pst_13661. The chlorotic mosaic symptoms were photographed 10 days after inoculation with barley stripe mosaic virus. The disease phenotypes were photographed 14 days after inoculation with *Pst* CYR31 on the fourth leaves. (b) *Pst* biomass was detected at 120 hpi in the silenced plants. *TaEF‐1α* and *PstEF‐1α* were used to normalize the RNA level of wheat and *Pst*, respectively. Mean and standard deviations were calculated from three independent replicates. Asterisks indicate significant difference in samples with *Pst_13661* silenced by HIGS in comparison with the control (*P* < 0.05, Student’s *t*‐test). (c) Representative images from *Pst_13661*‐knock‐down wheat were obtained with CellSens Entry software at 24 and 48 hpi. *Pst* hyphae were stained and then observed with an Olympus BX‐51 microscope (ocular: 10×; objective: 20×). H, haustoria; IH, infection hyphae. Bar = 50 μm. (d) The development of *Pst* was assessed as hyphal length and infection areas at 24 and 48 hpi. Standard deviation and means were calculated from three independent biological replications. Asterisks indicate significant difference compared to the sample of BSMV:00 (*P* < 0.05, Student’s *t*‐test).

Due to the practicality of BSMV (barley stripe mosaic virus)‐mediated HIGS (host‐induced gene silencing) assay for analysing the function of pathogen genes, we silenced *Pst_13661* with specific fragment. Ten days after infection with BSMV on the second leaves, mild chlorotic mosaic symptoms appeared on the fourth leaves in BSMV‐inoculated plants (Figure 3a). The photobleaching in *TaPDS*‐knock‐down plants indicated that the BSMV functioned in the plant. Fourteen days after inoculation with *Pst*, the growth and development of *Pst* were compromised in *Pst_13661* knock‐down plants (Figure 3a). The fungal biomass significantly decreased at 120 hpi in *Pst_13661* knock‐down plants (Figure 3b). The length of hyphae and infection areas was reduced compared with the control plants inoculated with BSMV:00 (empty vector) (Figure 3c and d). The transcript of *Pst_13661* in silenced plant was reduced significantly to 30%–40% at 48 and 120 hpi (Figure S4a). Due to the high similarity between *Pst_13661*, *Pst_13662* and *Pst_13645* in the polysaccharide deacetylase family, we also detected the silencing efficiency of *Pst_13662* and *Pst_13645*, and the expression of *Pst_13662* and *Pst_13645* decreased to 60%–70% at 48 and 120 hpi (Figure S4b and c). These results suggested that silencing of *Pst_13661* suppressed the growth and development of *Pst*.

### Transgenic wheat expressing Pst_13661 small RNA confers resistance to *Pst*

**Figure 4:**
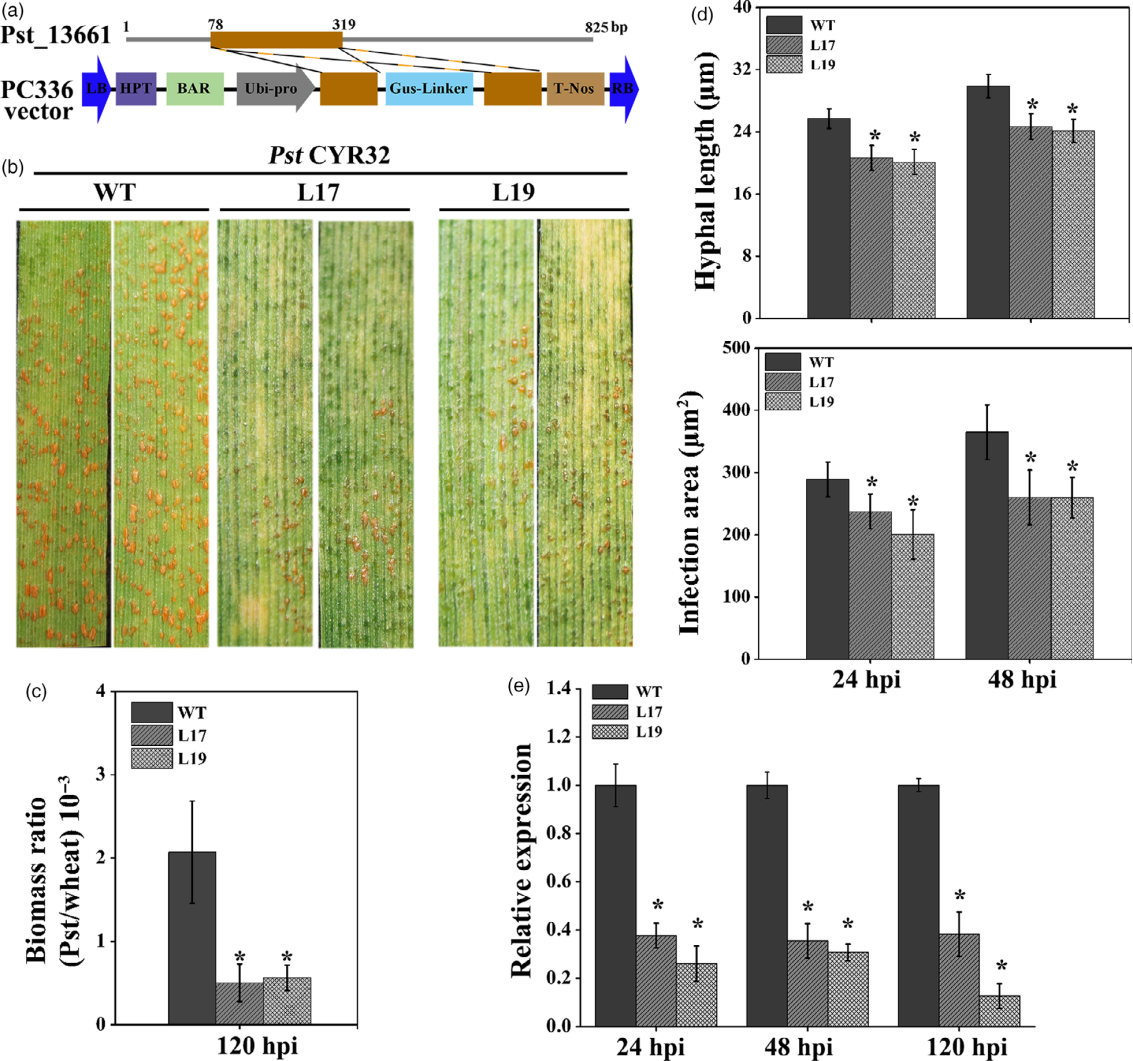
Small RNA‐mediated silencing of *Pst_13661* impairs pathogenicity of *Pst* CYR32 in transgenic wheat. (a) Diagram of RNAi cassette in the construct PC336 for silencing Pst_13661 in transgenic plant. LB, left border; HPT and Bar, the selectable marker genes; Ubi‐pro, ubiquitin promoter; T‐Nos, terminator; RB, right border. (b) The disease phenotype was captured at 14 days after inoculation with *Pst* race CYR32 on the second leaves of transgenic plant (L17 and L19). (c) *Pst* biomass was estimated by qRT‐PCR. *TaEF‐1α* and *PstEF‐1α* were used to normalize the RNA level of wheat and *Pst*, respectively. Mean and standard deviations were calculated from three independent replicates. Asterisks indicate significant difference (*P* < 0.05, Student’s *t*‐test) in samples with *Pst_13661* silenced by HIGS in comparison with the control. (d) Hyphal length and infection areas were assessed at 24 and 48 hpi. Standard deviation and mean were calculated from three independent biological replications. Asterisks indicate the significant difference compared to the sample of WT (*P* < 0.05, Student’s *t*‐test). (e) The silencing efficiency of *Pst_13661* was calculated by qRT‐PCR. Mean and standard deviation were calculated from three biological replicates. Asterisks indicate significant differences (*P* < 0.05) using Student’s *t*‐test.

To further evaluate the effect of *Pst_13661* on the pathogenicity of *Pst*, the T_4_ generation of transgenic wheat (L17 and L19) expressing short interfering RNAs (siRNAs) of *Pst_13661* was planted in the glasshouse (Figure 4a). To identify the resistance of the transgenic plant to *Pst*, *Pst* CYR32 was inoculated onto the second leaves of transgenic plants L17, L19 and wild‐type wheat (WT). After 14 days postinoculation, the transgenic plant expressed *Pst_13661*‐specific siRNAs with only a few uredia, while numerous uredia appeared on WT plants, indicating that the transgenic plant of *Pst_13661* significantly weakened the pathogenicity of *Pst* (Figure 4b). The fungal biomass in the transgenic plant also decreased to 40% compared with that in WT plant at 120 hpi (Figure 4c). Histological observations showed that the hyphal length was about 19 μm and infection areas were about 190 μm^2^ in transgenic wheat (L17 and L19), which were smaller than those (hyphal length:26 μm; infection areas: 290 μm^2^) in wild‐type plants at 24 hpi (Figure 4d). At 48 hpi, the infection areas were significantly reduced (220 μm^2^) than that in WT plants (380 μm^2^) and the hyphal length (23 μm) was also significantly shorter in transgenic plants compared with those (30 μm) in WT plant (Figure 4d). We also detected the accumulation of ROS and expression of defence genes in transgenic wheat. Histological observations of ROS showed that the accumulation of ROS was significantly increased at 24 and 48 hpi (Figure S5a and b). The transcript levels of *TaPR1* and *TaPR2* were increased to threefold to fivefold compared with that in WT plants (Figure S5c). For the detection of silencing efficiency, the transcript of *Pst_13661* in transgenic plants (L17 and L19) was reduced significantly to 20%–40% at 24 hpi, 48 hpi and 120 hpi (Figure 4e). Due to the small RNAi target of *Pst_13661* from the conserved polysaccharide deacetylase domain, we assessed the possibility of silencing other homologous genes in the pathogen. Although there were no continuous 21 nucleotides in homologous genes (Figure S6a), we also determined the silencing efficiency of the two genes (*Pst_13662* and *Pst_13645*) and found that the expression of *Pst_13662* decreased to 50% and the transcript of *Pst_13645* did not change compared with WT plants (Figure S6b).

To assess the durable disease‐resistant germplasm of transgenic wheat, we also identify the disease resistance to different *Pst* epidemic races from China. The amplification results of *Pst_13661* indicated that Pst_13661 was a conserved gene in the genomes of *Pst* races CYR31, CYR32 and CYR33 (Figure S7). Then, we inoculated transgenic RNAi plants with other main *Pst* races CYR31 and CYR33. The transgenic plants of *Pst_13661* were compromised the development of *Pst* races CYR31 and CYR33 (Figure S8a and b). The silencing efficiency of *Pst_13661* and *Pst_13662* reached 70%–80% and 30%–50%, respectively (Figure S8c and d). These results showed that knocking down *Pst_13661* in transgenic plants significantly weakened the pathogenicity of the *Pst* race CYR31, CYR32 and CYR33.

### Pst_13661 forms a homo‐polymer *in vivo*

**Figure 5:**
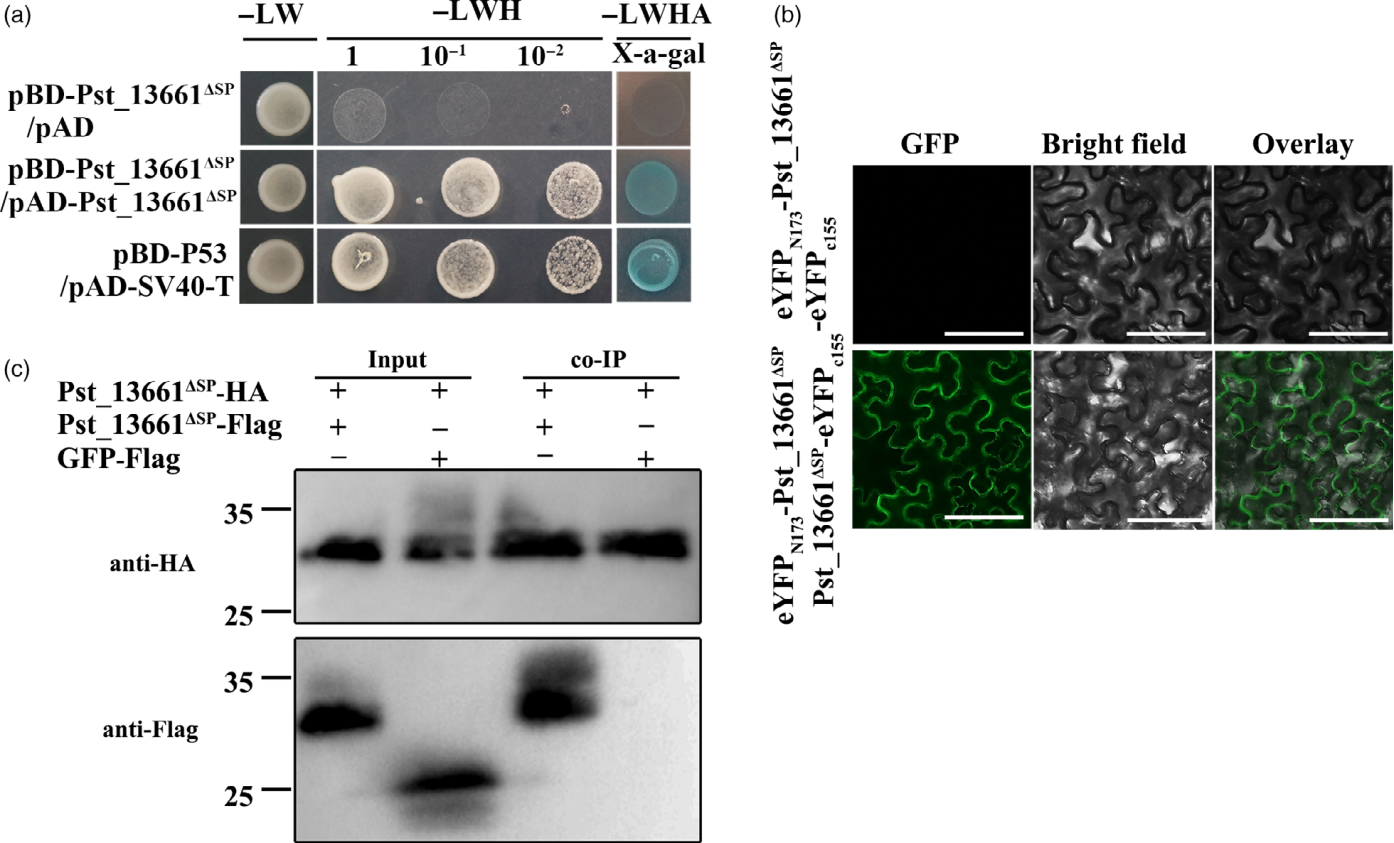
Pst_13661 forms homo‐polymer. (a) Yeast two‐hybrid analysis indicates that Pst_13661 interacts with itself. The transformants grew on the SD‐LW, SD‐LWH and SD‐LWHA with X‐a‐gal. L, leucine; W, tryptophan; H, histidine; A, adenine. (b)Bimolecular fluorescence complementation (BIFC) confirmed the interaction *in planta*. The eYFP‐Pst_13661 and the empty vector (the C‐terminal of YFP) were used as negative controls. Bar = 50 μm. (c) Co‐immunoprecipitation assay confirmed these interactions *in planta*. Anti‐HA and anti‐Flag were used to detect protein expression.

Due to the cluster distribution and high similarity among polysaccharide deacetylases, we inferred that Pst_13661 might function as homo‐polymers. To verify this hypothesis, we co‐transformed pBD‐Pst_13661 and pAD‐Pst_13661 in yeast. The transformed strains grew on both SD (‐Leu/‐Trp) and SD (‐Leu/‐Trp/‐His) media, as did the positive control (pBD/p53 and AD/SV40‐T; Figure 5a), indicating that Pst_13661 interacted with itself in yeast. These interaction relationships were further confirmed on the SD (‐Leu/‐Trp/‐His/‐Ade) plates with X‐a‐gal (Figure 5a). To further confirm that Pst_13661 forms homo‐polymers, bimolecular fluorescence complementation (BIFC) was utilized in *N*. *benthamiana*. *Pst_13661* was cloned into pSPYNE(R)173 and pSPYCE(M). The fluorescence signals of interaction between Pst_13661 filled the entire cell in the BIFC assay (Figure 5b). We further identified the homo‐polymer of Pst_13661 by co‐immunoprecipitation (co‐IP). Pst_13661^ΔSP^ were fused with Flag and HA tag, and co‐expressed in tobacco leaves. Total proteins were incubated with anti‐HA magnetic beads. We found that Pst_13661^ΔSP^‐Flag were pulled down by Pst_13661^ΔSP^‐HA on the beads (Figure 5c). On the contrary, the GFP‐Flag was not pulled from the total proteins (Figure 5c). The result of co‐IP assay indicates that Pst_13661 forms homo‐polymers in *vivo*.

### Pst_13661 accumulates mainly in the apoplast and have affinity to chitin

**Figure 6:**
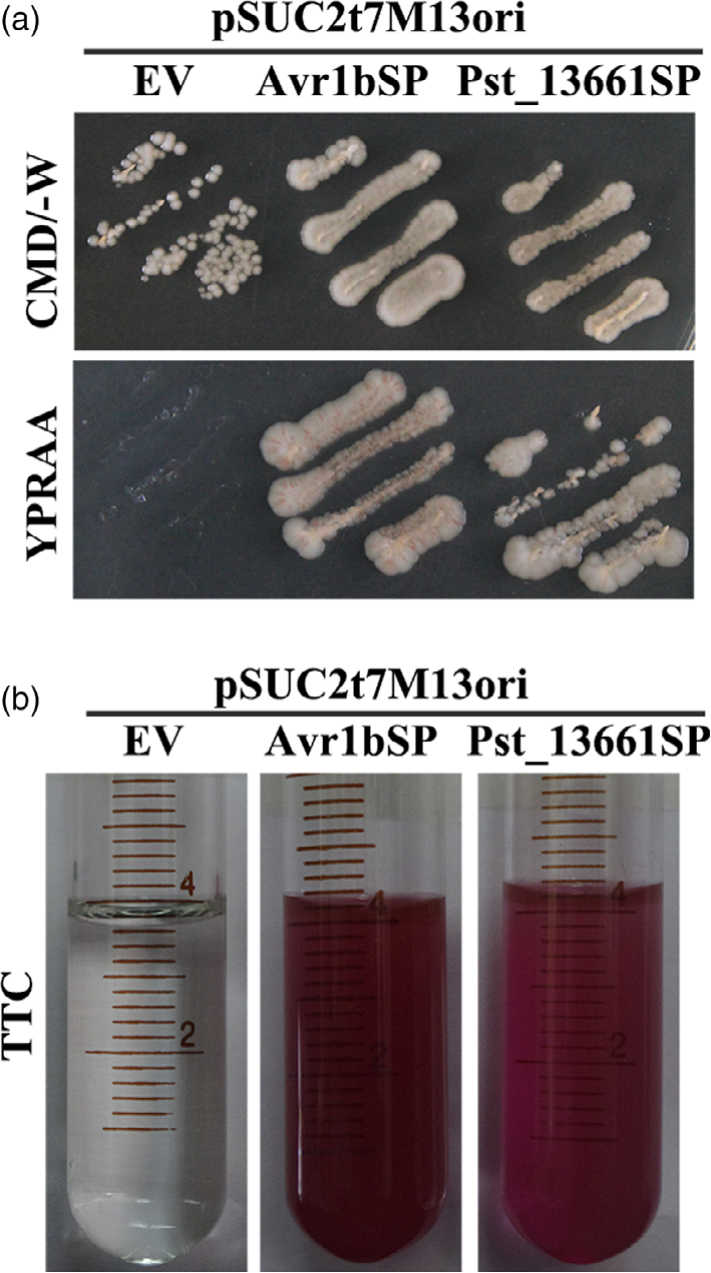
The functionality of the Pst_13661 signal peptide. (a) The secretion function of the signal peptide of Pst_13661 was confirmed by yeast secretion system. Yeast YTK12 strains carrying the empty vector, the signal peptide of Pst_13661 and Avr1b grew on the CMD‐W and YPRAA plates, respectively. (b) The enzymatic activity of invertase was detected by the reduction of 2, 3, 5‐triphenyltetrazolium chloride (TTC) to insoluble red‐coloured 1, 3, 5‐triphenylformazan (TPF). The negative control is the YTK12 strain carrying empty vector, and the positive control is the YTK12 strain carrying the signal peptide of Avr1b.

To test the function of the signal peptides, the enzyme invertase was used to identify their function in yeast. The stain YTK12 with its endogenous invertase gene deleted and one vector pSuc2t7M13ori possessing an invertase gene without its signal peptides (Jacobs *et al.*, 1997). Pst_13661SP (the signal peptide) was inserted into the vector pSuc2t7M13ori and transformed into YTK12 stain. Transformants grew on the complete minimal medium lacking tryptophan (CMD‐W), indicating that recombinant plasmids and the empty vector pSuc2t7M13ori were transformed into YTK12 strain (Figure 6a). Like the positive control Avr1bSP, Pst_13661SP grew well on the YPRAA plates. But the empty vector lacking the corresponding signal peptides did not grow on the selective medium (Figure 6a). We also detected the enzyme activity of the secreted invertase in medium and found that 2,3,5‐triphenyltetrazolium chloride (TTC) was converted into insoluble red‐coloured 1,3,5‐triphenylformazan (TPF) in the presence of invertase, indicating that transformants with Avr1bSP and Pst_13661SP secreted invertase into the medium (Figure 6b).

**Figure 7:**
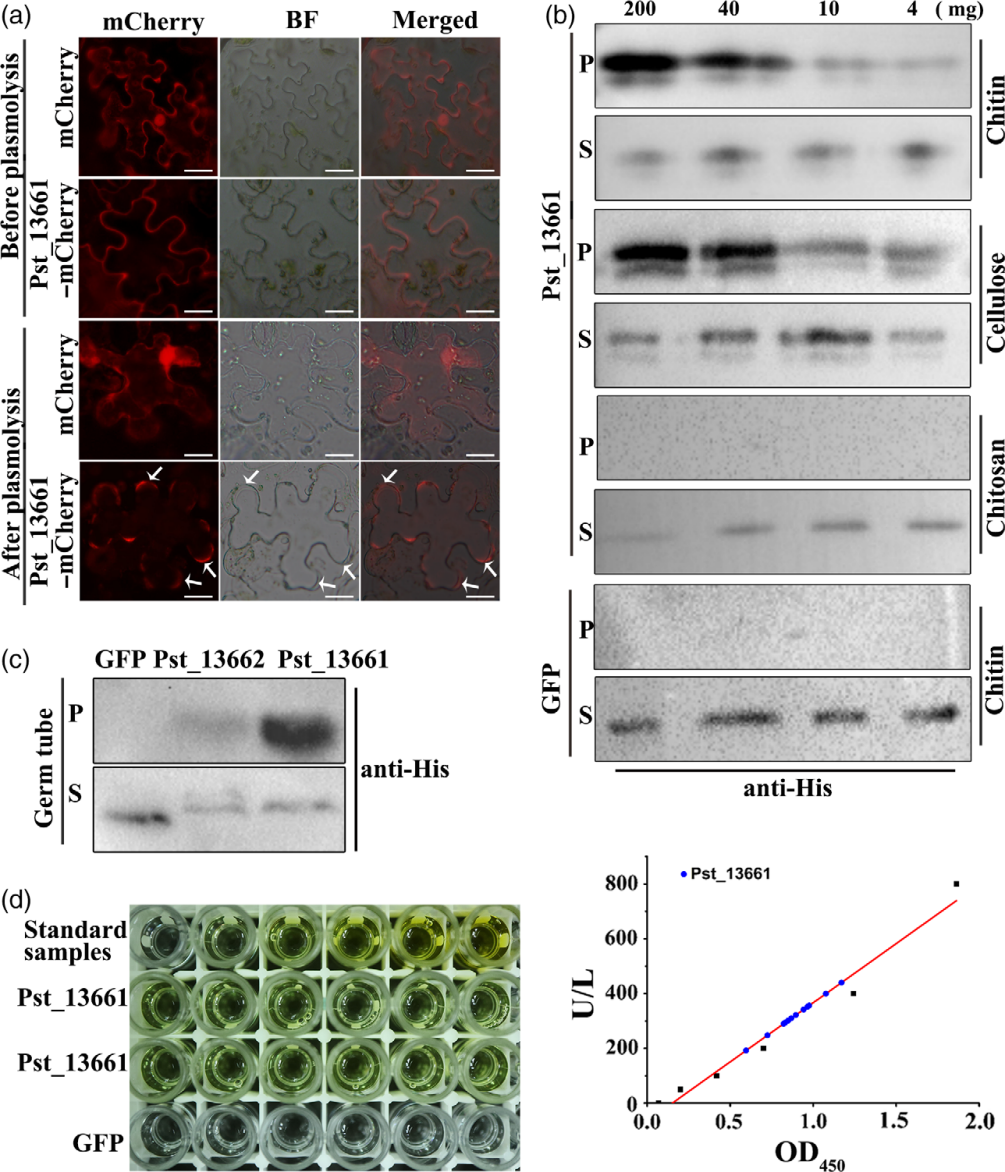
Pst_13661 accumulates in the apoplast and has affinity to chitin. (a) Transiently expressed Pst_13661‐mCherry localized in the apoplast of *N*. *benthamiana*. Plasmolysis was induced by 50 mm NaCl. Arrows represent plasmolysis regions. BF, bright field, Bar = 20 μm. (b) Pst_13661 has high affinity to chitin and cellulose. Pst_13661 or GFP protein was diluted and incubated with different concentrations of affinity materials at 4 °C for 4 h. Samples of the supernatant and affinity precipitate were detected with anti‐His by Western blot. GFP protein was a negative control. S, supernatant; P, affinity precipitation. Different concentrations of affinity materials are 200 mg, 40 mg, 10 mg and 4 mg. (c) Pst_13661 protein binds the fungal cell wall. Pst_13661, Pst_13662 or GFP proteins were diluted and incubated with germ tubes of *Pst* at 4°C for 4 h. The supernatant and affinity precipitate were detected with anti‐His by Western blot. GFP protein was a negative control. S, supernatant; P, affinity precipitation. (d) Deacetylase enzyme activity of Pst_13661 (100 µg/mL) was detected with the chitin deacetylase (CDA) ELISA kit. The left panel indicates the colour change in substrate solution in the presence of Pst_13661 protein and standard samples. GFP was a negative control. Standard samples were 0, 50, 100, 200, 400, and 800 U/L. The right panel shows the standard curve of deacetylase enzyme activity according to the dilution of standard samples. The blue circle represents Pst_13661.

To understand its localization, we first predicted that Pst_13661 was an apoplastic protein based on analysis with ApoplastP software (Table S1). Because of the presence of a signal peptide in the protein (Figure S9a), we used the full length of Pst_13661 tagged with mCherry and detected the presence of Pst_13661 in the apoplast after sucrose‐induced plasmolysis (Figure 7a). However, mCherry alone was not detected in the apoplast region (Figure 7a). These results indicate that Pst_13661 is secreted into apoplast region.

Polysaccharide deacetylases have been considered important in protecting pathogen hyphae from recognition and degradation by host‐degrading enzymes in the apoplast. Due to the polysaccharide deacetylase domain of Pst_13661, we tested its ability to bind to polysaccharides. Chitin, cellulose and chitosan were chosen for affinity precipitation with different concentrations (van den Burg *et al.*, 2006). The affinity precipitation confirmed that Pst_13661 had high affinity to chitin and cellulose compared with the negative control GFP, which did not bind to chitin, cellulose or chitosan (Figure 7b). To further confirm the ability to bind to the fungal cell wall, germ tubes were incubated with Pst_13661, Pst_13662 and GFP protein. Pst_13661 was significantly precipitated by germ tubes of *Pst*, but like the negative control GFP, Pst_13662 was not enriched (Figure 7c). Sequence alignment of PDAs indicated that Pst_13661 contained five motifs in the polysaccharide deacetylase domain and these motifs were conserved compared to the characterized PDAs or CDAs from other fungal species (Figure S9a and b). The deacetylase activity of Pst_13661 was also detected with the chitin deacetylase ELISA kit which revealed the colour change in the substrate solution in the presence of Pst_13661 protein (Figure 7d). These results suggest that Pst_13661 may function as a deacetylase to bind fungal cell wall in the apoplast for pathogenicity.

### Pst_13661 suppresses chitin‐induced plant immunity

**Figure 8:**
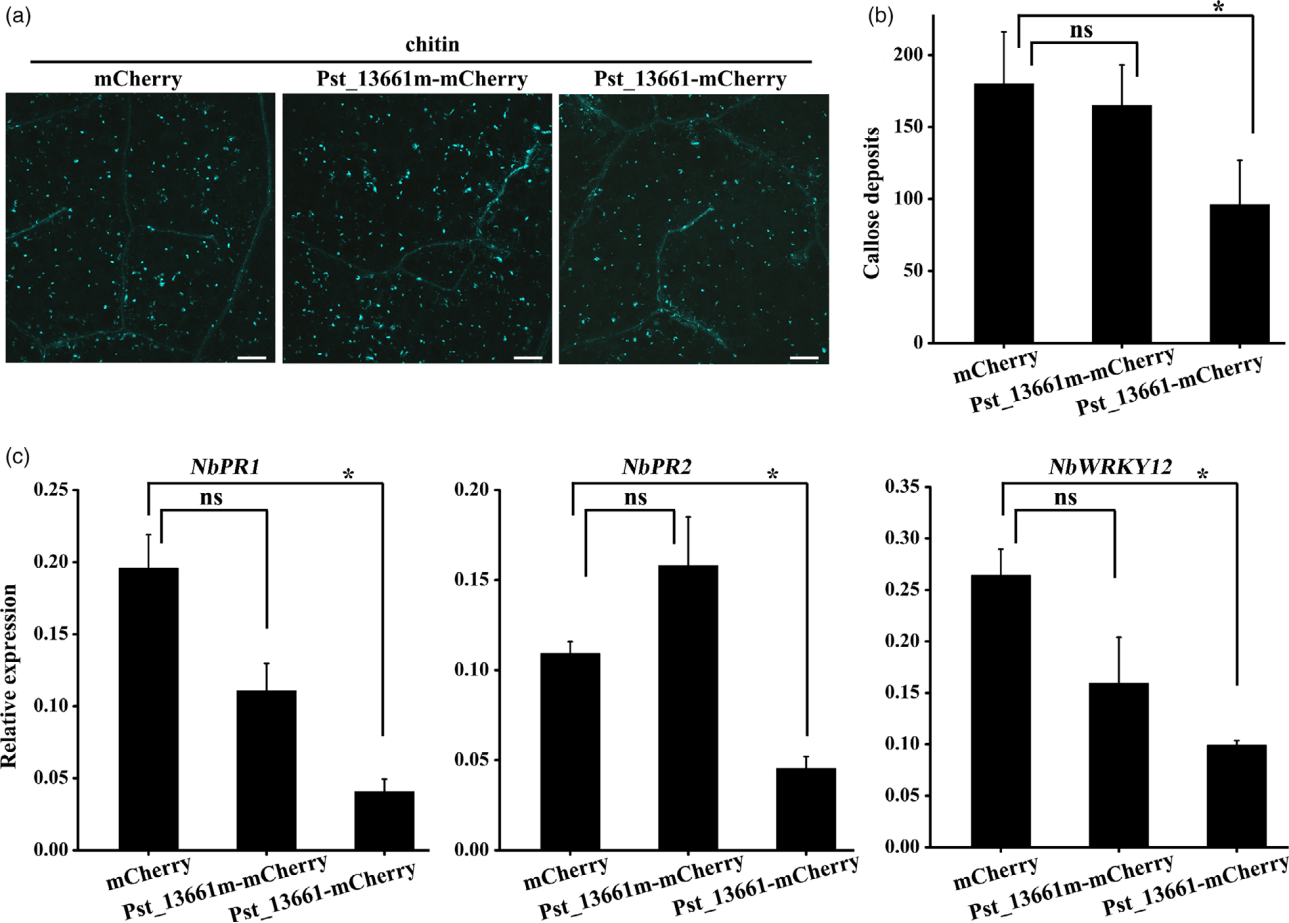
Pst_13661 suppresses chitin‐induced plant defence in *N*. *benthamiana*. (a) Callose deposition induced by 200 µg/mL chitin in tobacco leaves transiently expressing Pst_13661: mCherry, Pst_13661m: mCherry or mCherry alone. Images were obtained 24 h after infiltration with chitin. Bar = 200 μm. (b) Pst_13661 suppresses callose spots in *N*. *benthamiana*. The number of callose spots per 1‐mm^2^ was assessed with the ImageJ software. Mean and standard deviations were calculated from three biological replicates. Asterisks mark significant difference based on Student’s *t*‐test (*, *P* < 0.05; ns, not significant). (c) The expression levels of *PR1*, *PR2* and *WRKY12* in *N*. *benthamiana* leaves transiently expressing Pst_13661: mCherry, Pst_13661m: mCherry or mCherry after infiltration with chitin were assayed by qRT‐PCR with *Act1* as a reference gene for normalization. Mean and standard deviations were calculated from three biological replicates. Asterisks indicate significant differences based on Student’s *t*‐test (*, *P* < 0.05; ns, not significant).

To test whether Pst_13661 could suppress chitin‐induced plant immunity, Pst_13661‐mCherry, Pst_13661m‐mCherry (the mutant of Pst_13661) and mCherry first were transiently expressed in *N*. *benthamiana*, respectively. All of the active enzyme sites in five conserved motifs were destroyed in mutant Pst_13661m (Figure S9c) according to the enzymatic characteristics of PDAs (Gao *et al.*, 2019). The leaves were treated with 200 µg chitin/mL after 48 h postinoculation with *Agrobacterium,* and at 24 h postinfiltration with chitin, aniline blue staining showed less callose accumulation in *N*. *benthamiana* leaves expressing Pst_13661‐mCherry than in leaves expressing mCherry alone and Pst_13661m‐mCherry (Figure 8a). Pst_13661‐mCherry suppressed chitin‐induced callose accumulation, but Pst_13661m‐mCherry and mCherry could not (Figure 8b). The marker genes of plant immune in leaves expressing Pst_13661‐mCherry were also detected. The transcript levels of *PR1*, *PR2* and *WRKY12* were 4‐, 2‐, and 2‐fold lower, respectively, in tobacco leaves expressing Pst_13661:mCherry than in the control plants expressing mCherry alone (Figure 8c). But in leaves expressing Pst_13661m‐mCherry, the expression of defence genes was not significantly different compared with the control (Figure 8c). These results indicate that Pst_13661 suppresses chitin‐induced plant immunity.

## Discussion

During plant‐pathogen interactions, the cell wall at the hyphal tip of fungal pathogens is exposed to a complex environment with diverse degradative proteases in the plant apoplast. Plant chitinase releases a mass of chitin fragments such as chitin oligomers to activate the plant immunity against the invading pathogen (Ramonell *et al.*, 2005; Wan *et al.*, 2008a; Wan *et al.*, 2008b). On the contrary, polysaccharide/chitin deacetylases secreted from fungi into the intercellular matrix are capable of removing acetyl groups from chitin to chitosan (Baker *et al.*, 2011). The conversion of chitin to chitosan protected against degradation of fungal hyphae and the recognition of plant receptors, and also chitosan could prevent the generation of chitin oligomers during chitinase attack (Mauch *et al.*, 1988; Ride and Barber, 1990; Stegrist and Kauss, 1990). In this study, we identified a Pst_13661 of *Pst*; it predicted to be one polysaccharide deacetylase with a NodB homology domain of the carbohydrate esterase family 4 (CE‐4) (Zhao *et al.*, 2010). Hence, we tested its ability to bind to polysaccharides, and the results showed that Pst_13661 had deacetylase enzyme activity and a higher affinity to chitin, cellulose and germ tubes of *Pst.* Besides, Pst_13661 suppressed chitin‐induced plant basal immunity, pointing to a role of polysaccharide deacetylases for the infection and colonization of rust pathogen. In addition, the transgenic wheat plants silencing of Pst_13661 showed an increased resistance to *Pst* with the reduction in the accumulation of ROS and expression of plant immune genes and delayed development of disease symptoms. Therefore, we speculated that Pst_13661 could bind to fungal hyphae and deacetylate chitin to chitosan on the cell wall for reduction in the degradation of cell wall and generation of chitin elicitor for the colonization of rust pathogen.

Chitin/polysaccharides deacetylases are conserved and exist in many fungi, insects and marine bacteria (Zhao *et al.*, 2010). Interestingly, there were more than 20 members of chitin/polysaccharides deacetylases in the *Pst* genome and about 10 members had high similarity and were distributed in five gene clusters. An example is *Pst_13661* and *Pst_13662*. The similarity of *Pst_13661* and *Pst_13662* reached 80% in nucleotide and amino acid sequences, perhaps because they might come from a common ancestral gene by gene duplication during the evolutionary process to protect against plant defence responses. Interestingly, *Pst_13661* had high expression levels during *Pst*–wheat interaction, but *Pst_13662* with a low expression level. In view of the difference in expression, we speculated that *Pst_13661* and *Pst_13662* had functional differentiation in *Pst*–wheat interaction. In other pathogens, such as *Clostridium difficile*, also possesses virulence gene clusters and putative pathogenicity islands in the genome (Gold *et al.*, 2001; Hacker and Kaper, 2000), which may be involved in pathogenicity during infection stages. In agreement with this, previous studies revealed that 12 clusters of genes encoding secreted proteins in *Ustilagomaydis* were induced in infected tissue, and in gene‐deletion plants, converted the disease reaction from susceptibility to resistance (Kamper *et al.*, 2006). The mig2 gene locus in *Ustilagomaydis* contains five highly homologous genes, mig2–1 to mig2–5, that are up‐regulated during infection stages (Basse *et al.*, 2002). Interestingly, this phenomenon also applies to plants. A tandem of subtilisin‐like protease genes in tomato (*P69A*, *P69B*, *P69C* and *P69D*), also termed pathogenesis‐related PR‐P69 protease, exhibited a high similarity to each other and were involved in protein degradation against pathogen infection (Jorda *et al.*, 1999; Tornero *et al.*, 1997). Therefore, we speculate that the gene clusters of polysaccharides deacetylases may contribute to pathogenicity of *Pst*.

Stripe rust caused by *Puccinia striiformis* f.sp. *tritici* (*Pst*) is a major disease causing significant yield losses and threatening the security of wheat worldwide. The most effective approach to controlling the disease is worthy of consideration and further investigation. However, the use of traditional fungicides to reduce wheat rust or other diseases not only adds a substantial production cost but also negatively influences the environment. Although a few new fungicides to control many crop diseases have been discovered, the prospect of applying non‐polluting agents does not seem promising in the foreseeable future. To date, the stripe rust resistance genes *Yr1*, *Yr2*, *Yr6*, *Yr7*, *Yr9* and *Yr10* and other genes (about 78‐Yr genes) and 67 temporarily designated Yr genes were found in multiple wheat varieties in previous studies (Chen and Kang, 2017). These resistance genes will be expected to play a role in wheat rust control and prevention in the future. But, generally speaking, many race‐specific resistance genes are effective for only 3–5 years, indicating a serious challenge to discover genetic resources of resistance (Chen, 2005). Especially, due to the ill‐advised planting distribution of resistant cultivars coupled with the rapid evolution and variation of the pathogen usually results in the loss of favourable resistant varieties of cereal crops and outbreaks of wheat rust. In 1999, the new race Ug99 caused the loss of wheat resistance gene Sr31 and widespread susceptibility of 90% of wheat‐growing areas, thus threatening the worldwide crop security (Pretorius *et al.*, 2012; Singh *et al.*, 2011). Hence, developing broad‐spectrum resistance wheat materials is urgent for sustainable control of the rust disease. In this study, we generated RNAi transgenic wheat plants using the polysaccharide deacetylases of *Pst*, which are highly resistant to major epidemic isolates and exhibit broad‐spectrum resistance to *Pst*. This study provides a clue that exploring key pathogenicity genes of the pathogen is an alternative potential strategy to achieve durable disease control.

The ultimate goal of basic research on pathogenesis is to prevent the occurrence of disease or reduce the severity and improve production practices. The study proved that the polysaccharide deacetylase Pst_13661 affected the type of chitin from the pathogen cell wall and weakened the virulence of main epidemic *Pst* races in transgenic wheat plants. Hence, perhaps control of wheat rust will become feasible by identifying antifungal targets to chitosan, chitin deacetylases or other important virulence factors. Thus, this study provides new insight for better understanding and management of wheat rust.

## Experimental procedures

### Biological material, culture condition and fungal inoculation

*Escherichia coli* DH5a and *Agrobacterium tumefaciens* GV3101 for bacterial transformation were cultured on Luria‐Bertani (LB) medium at 37 and 28 °C, respectively. Yeast strains AH109 and YTK12 were grown at 30 °C for protein interaction assay and secretion assay, respectively. For plant materials, *N*. *benthamiana* and wheat seedlings were grown in a growth chamber at 22 and 16 °C with 8/16‐h night/day, respectively. For rust inoculation, urediniospores of the virulent race CYR31, CYR32 and CYR33 were produced on the second leaves of wheat cultivar Suwon 11 as described previously (Kang *et al.*, 2002).

### Genetic transformation of wheat

The wheat cultivar used for genetic transformation was the hexaploid wheat cultivar CB037, which was susceptible to *Pst* race CYR31, CYR32 and CYR33. A 243 bp of specific fragment of Pst_13661 was amplified using forward primer 5′‐ACACAAATGATATCCGCACCTT‐3′ and reverse primer 5′‐AATGGCCGTTGTTGAATCTTT‐3′ (Table S2). Then, this fragment was inserted into PC336 (*Ubi:GWRNAi:Nos*) plasmid using gateway cloning method. About 1500 isolated wheat embryos were cultured and bombarded using the PDS‐1000/He Particle Delivery System (Bio‐Rad Laboratories, CA, USA) according to the standard methods as previously described (Lv *et al.*, 2014). Regeneration and selection of wheat embryos were selected using in the corresponding medium with 3 mg/L bialaphos, finally 2 putative transgenic plants (L17 and L19) were identified by the positive amplification in Shandong agricultural university, and the primers were used in Table S2. The two independent transgenic lines L17 and L19 were planted in a glasshouse to rapid propagation and were inoculated *Pst* races for the identification of disease phenotype.

### Sequence analysis

DNA sequences of *Pst_13661* and *Pst_13662* were amplified from the genomic DNA of *Pst* race CYR31, CYR32 and CYR33 and purified by the CTAB method (Healey *et al.*, 2014). The open reading frame (ORF) of *Pst_13661* was derived from cDNA of CYR32 purified by RNA Purification Kit (QIAGEN). Analysis of protein domains is based on HMMER software (http://www.ebi.ac.uk/Tools/hmmer/). The sub‐localization was predicted by ApoplastP and TargetP. The molecular weight of *Pst_13661* was predicted by the Compute pI/Mw tool (http://web.expasy.org/compute_pi/). The sequence alignments were carried out with DNAMAN software (version 6). Conserved domain structures of polysaccharide deacetylases were analysed using InterProScan software. According to the conserved core domains of the polysaccharide deacetylase family, the sequence was obtained from BLASTX, and the phylogenetic relationship was established by Neighbour‐Joining (NJ) methods in program MEGA (version 5).

### Plasmid constructs

For plasmid constructs for the silencing system, the small segment of *Pst_13661* was inserted into the virus plasmid *γ*. For yeast two‐hybrid system, *Pst_13661^ΔSP^* was linked into pGBKT7 and pGADT7 at *EcoRI* and *BamHI* sites, respectively. *Pst_13661* was inserted into pICH86988 vector with mCherry tag for sub‐localization and linked into pSPYNE(R)173 and pSPYCE(M) for bimolecular fluorescence complementation. *Pst_13661^ΔSP^* was inserted into pICH86988 vector with HA and Flag tag for co‐immunoprecipitation. All primers used in this study are listed in Table S2.

### RNA extraction and qRT‐PCR

Wheat leaves infected with *Pst* were harvested at 6, 12, 20, 24, 36, 48, 72 and 96 h postinoculation (hpi) for the detection of the transcript levels of *Pst_13661* and *Pst_13662* during the *Pst*–wheat interaction (Cheng *et al.*, 2015). Samples of knock‐down plants infected with *Pst* were extracted at 24, 48 and 120 hpi to determine the silencing efficiency of *Pst_13661*. Urediniospores and infected leaves were ground in liquid nitrogen, and RNA was isolated using MiniBEST Plant RNA Extraction Kit (TaKaRa) following the manufacturer’s instructions. DNA was extracted from urediniospores of *Pst* CYR32 using CTAB method for sequence amplification (Healey *et al.*, 2014). qRT‐PCR was carried out using Bio‐Rad CFX Manager (version 3.1) under the following conditions: 95 °C for 10 min to preheat, 40 cycles at 95 °C for 10 s, 56 °C for 20 s to calculate cycle threshold values, followed by 95 °C for 20 s, 60 °C for 1 min and 95 °C for 15 s. *Pst_EF* and *Ta_EF* were used as the internal reference genes for qRT‐PCR. The double standard curves of absolute quantitation were from the fold dilution of wheat and *Pst* cDNA with 10×, 20×, 50×, 100×, 200×, 500×, 1000×. The correlation coefficients for curves were above 0.99, and the slope was approximately −3.3 (Panwar *et al.*, 2013).

### Host‐induced gene silencing in wheat

For transiently silencing *Pst_13661,* a 300‐bp‐specific fragment of *Pst_13661* served as the RNAi target using BSMV‐HIGS system (Holzberg *et al.*, 2002; Nowara *et al.*, 2010). Then, corresponding recombinant vectors (*TaPDS‐γ*, *Pst_1366*1*‐γ*), α, β and γ were linearized by corresponding enzymes and transcribed into RNA. Then, the BSMV RNA of α and β was mixed with *TaPDS‐γ*, *Pst_1366*1*‐γ* and γ in FES buffer, respectively. The second leaves of wheat seedings were inoculated with the BSMV RNA mixture and maintained in the glasshouse at 25–27 °C for 10 days. Then, the fourth leaves of plants inoculated with BSMV RNA were inoculated with urediniospores of *Pst* CYR31 and samples were harvested at 24, 48 and 120 hpi. The phenotypes were recorded, and representative photographs were captured at 14 dpi. For the identification of transgenic plants, urediniospores of *Pst* CYR31, CYR32 and CYR33 were inoculated on the second leaves of the wheat cultivar CB037 and samples harvested at 24 hpi for silencing efficiency.

### Protein expression in *N*. *benthamiana*

For Bax suppression assay, the recombinant vector (PVX:Pst_13661:HA, PVX:eGFP:HA, PVX:Avr1b:HA and PVX:Bax) was transformed into *A*. *tumefaciens* GV3101, respectively. The infection of *N*. *benthamiana* followed procedures described previously (Wang *et al.*, 2011). Briefly, the recombinant strains were washed three times with 10 mm MgCl_2_ and infiltrated into leaves of 4‐week‐old *N*. *benthamiana* at an OD_600_ of 0.4. The strains containing GFP, Avr1b and Pst_13661 were infiltrated into leaves 24 h prior to infiltrating the strain containing Bax. The infected leaves were harvested for protein extraction at 72 hpi. For localization, the full length of Pst_13661 tagged mCherry was transformed into *A*. *tumefaciens* GV3101. Then, the bacterial resuspension with OD_600_ 0.6 was injected into 4‐week‐old tobacco leaves with a syringe and kept in the glasshouse at 22 °C. At 48 hpi, the epidermis of injected leaves was removed and observed by fluorescence microscopy. For plasmolysis, the epidermis of injected leaves was treated with 50 mm NaCl for 10–20 min and then observed by fluorescence microscopy. For interaction by BIFC assay, Pst_13661^ΔSP^ was inserted into pSPYNE(R)173 and pSPYCE(M) and transformed into *A*. *tumefaciens* GV3101 (Waadt *et al.*, 2008). *Agrobacterium* strains were infiltrated at an OD_600_ of 0.5. Three days after inoculation, the pictures were captured by confocal microscopy with 488‐nm laser. For chitin‐induced plant immunity, *Agrobacterium* strains containing Pst_1366‐mCherry, Pst_13661m‐mCherry and mCherry were infiltrated at an OD_600_ of 0.6, respectively. Chitin was infiltrated at 48 h postinoculation with *Agrobacterium.* Samples were harvested at 12 h postinfiltration with chitin for detection of callose deposits and at 3 h for detection of defence genes.

For co‐immunoprecipitation, Pst_13661^ΔSP^‐HA and Pst_13661^ΔSP^‐Flag were transformed into *A*. *tumefaciens* GV3101 and *Agrobacterium* strains carrying Pst_13661^ΔSP^‐HA and Pst_13661^ΔSP^‐Flag were infiltrated at an OD_600_ of 0.5:0.5. Three days after inoculation, the total proteins of *N*. *benthamiana* were extracted with native lysis buffer (Cat No R0030, Solarbio Life Sciences). The total extracted proteins were centrifuged at 15 000 ***g*** for 15 min, and the supernatant was transferred into a fresh tube for co‐immunoprecipitation assay. Anti‐HA magnetic beads (Cat No 88836, Thermo Fisher Scientific) were washed three times with 1000 µL of extraction buffer and incubated with the total protein solution at 4 °C for 2–3 h. The beads were collected and washed three times with 500 µL of 50 mm Tris‐HCl buffer, pH7.4, 150 mm NaCl and 0.5% Tween‐20. Proteins that bound to the magnetic beads were detected by Western blotting with anti‐HA and anti‐Flag, respectively.

### Transient expression of proteins in yeast strain

For interaction by yeast two‐hybrid system, Pst_13661^ΔSP^ (without signal peptide) (pBD‐Pst_13661^ΔSP^) and Pst_13661 (pAD‐ Pst_13661^ΔSP^) were transformed into yeast strain AH109 and grown on SD‐Trp medium. The corresponding transformants were diluted and selected on SD/‐Leu/‐Trp/‐His/‐Ade medium with X‐a‐gal at 30°C. For the function of signal peptide of Pst_13661, Pst_13661SP (signal peptide of Pst_13661) and Avr1bSP (signal peptide of Pst_13661) were inserted into plasmid pSuc2t7M13ori and transformed into yeast strain YTK12 (Jacobs *et al.*, 1997). Transformant strains were screened on plates of CMD/‐W medium and YPRAA medium. The invertase activity was detected by the reduction of 2, 3, 5‐triphenyltetrazolium chloride (TTC) to an insoluble, red‐coloured 1, 3, 5‐triphenylformazan (TPF). Transformants were cultured in liquid CMD/‐W medium at OD_600_ of 0.3, and approximately 1.5 mL of cell suspension was collected and re‐suspended with 250 μL of 10 mm acetic acid–sodium acetate buffer (pH 4.7), 500 μL of 10% sucrose solution (w/v) and 750 μL of sterile distilled water at 37 °C for 10 min. After centrifugation at 12 000 ***g*** for 1 min, 400 μL of the supernatant was transferred into a glass test tube containing 3.6 mL of 0.1% TTC solution at room temperature for 5 min.

### Protein expression, purification and analysis

*Pst_13661^ΔSP^* was inserted into PET‐28a vector and transformed into strain BL21. The crude proteins of Pst_13661^ΔSP^ with His tag were purified by Ni‐chelating affinity chromatography (Cat No 17531802, General Electric Company). About 0.3 µg of proteins, chitin beads (Cat No S6651S, New England Biolabs), chitosan (Cat No 50494, Sigma‐Aldrich) and cellulose (Cat No 310697, Sigma‐Aldrich) at different concentrations (200, 40, 10 and 4 mg) were used for affinity precipitation (van den Burg *et al.*, 2006). The proteins and affinity materials were incubated at 4 °C for 4 h with 5 mL of incubation buffer (50 mm Tris/HCl, pH7.5, and 150 mm NaCl). After 4 h, the compounds were precipitated by centrifugation at 5000 ***g*** for 5 min and 100 µL of supernatant was stored for analysis. The precipitation washed five times with 1 mL of incubation buffer. The final precipitation and the supernatant were added into protein loading buffer for protein analysis by Western blot. For detection of deacetylase enzyme activity, Pst_13661 protein (about 100 µg/mL) was used with the chitin deacetylase (CDA) ELISA kit (Cat No RJ‐27869; Shanghai Renjie Biotechnology Co., Ltd) according to the manufacturer’s instructions.

### Histological observations

The wheat leaves from inoculated BSMV plants at 24 and 48 hpi and from transgenic plant at 24, 48 and 120 hpi were harvested for observations of fungal hyphae. These leaves were cut into several fragments, and the chlorophyll was removed in ethanol/acetic acid (1:1 v/v) as previously described (Cheng *et al.*, 2015). Then, these fragments were autoclaved at 121 °C for 5 min after they became transparent by treatment with chloral hydrate. Leaf segments were stained with WGA (wheat germ agglutinin) Alexa‐488 solution (Cat No W11261, Thermo Fisher Scientific) and observed with an Olympus BX‐51 microscope at 488 nm with CellSens Entry software (Olympus, Tokyo, Japan). Thirty infection sites on each randomly selected leaf segment were measured for hyphal length and infection area. To detect callose deposits, infected leaves were decolorized (absolute ethyl alcohol:acetic acid, 1:1 v/v) and immersed overnight in chloral hydrate. Transparent leaf segments were stained with 0.05% aniline blue in 0.067 m K_2_HPO_4_ (pH 9.6). Callose deposits were analysed in fields of 1 mm^2^ using ImageJ software.

## Conflict of interest

The authors declare no competing interests.

## Author contributions

X.W, Q.X, J.F.W and C.T conceived this research and Q.X conducted most of the experiments. Q.X, S.S and J.Z constructed the related vectors and cultured all tobacco and wheat plants. J.J.W and H.F.Z created the transgenic plant of Pst_13661. Q.X and S.S performed RNA extraction and protein‐protein interaction. J.Z and J.F.W purified the related proteins and transient expression of proteins. Q.X wrote this manuscript and X.W and Z. K revised it.

## Supporting information


**Figure S1** **.** DNA sequences of *Pst_13661*.

**Figure S2.** *Pst_13661* and *Pst_13662* are of high similarity.

**Figure S3.** Expression patterns of Pst_13661 and Pst_13662 at different stages infection.

**Figure S4.** The transcript levels of *Pst_13661*, *Pst_13662* and *Pst_13645* were measured in the silenced plants at 48 hpi and 120 hpi.

**Figure S5.** Detection of plant defense response in transgenic plant expressing siRNAs of Pst_13661.

**Figure S6.** Target of *Pst_13661* RNAi transgenic plants and calculation of the silencing efficiency.

**Figure S7.** *Pst_13661* are of high similarity in main epidemic *Pst* races.

**Figure S8.** Pathogenicity of main epidemic *Pst* races CYR31 and CYR33, is impaired in Pst_13661 RNAi transgenic wheat.

**Figure S9.** Conserved PDA motifs in Pst_13661.

**Table S1.** Characteristics of five pairs of highly similar gene clusters in *Pst* genome.

**Table S2.** Primers used in this study.
