# Supplementary figures and images for: A polysaccharide deacetylase from Puccinia striiformis f. sp. tritici is an important pathogenicity gene that suppresses plant immunity
Source: Plant Biotechnol J. 2020 Mar 4;18(8):1830–42. doi: 10.1111/pbi.13345 (PMC7336287; doi:10.1111/pbi.13345)

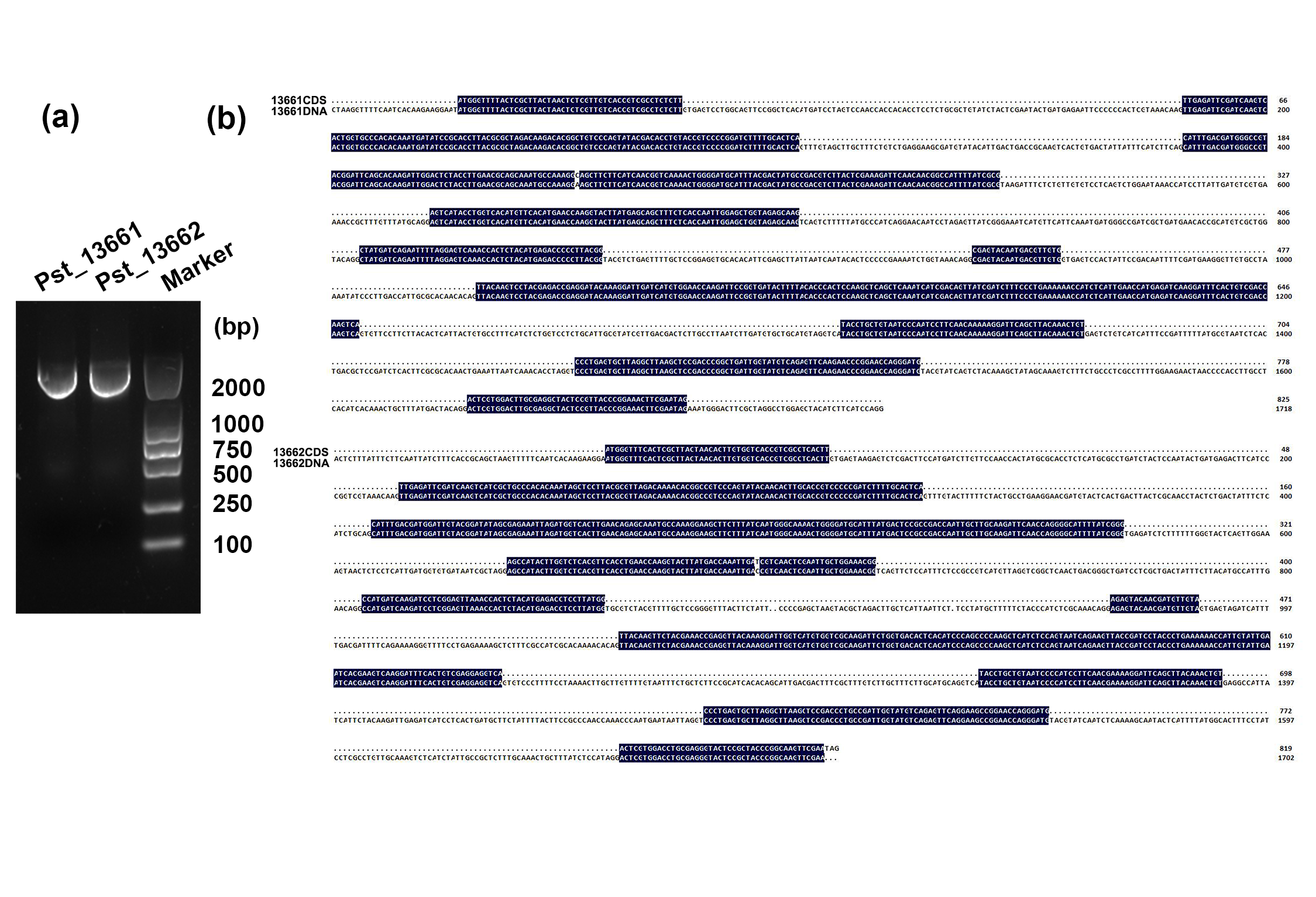

Supplement: Supplementary file 1 — Figure S1 . DNA sequences of Pst_13661. [file PBI-18-1830-s005.tif]

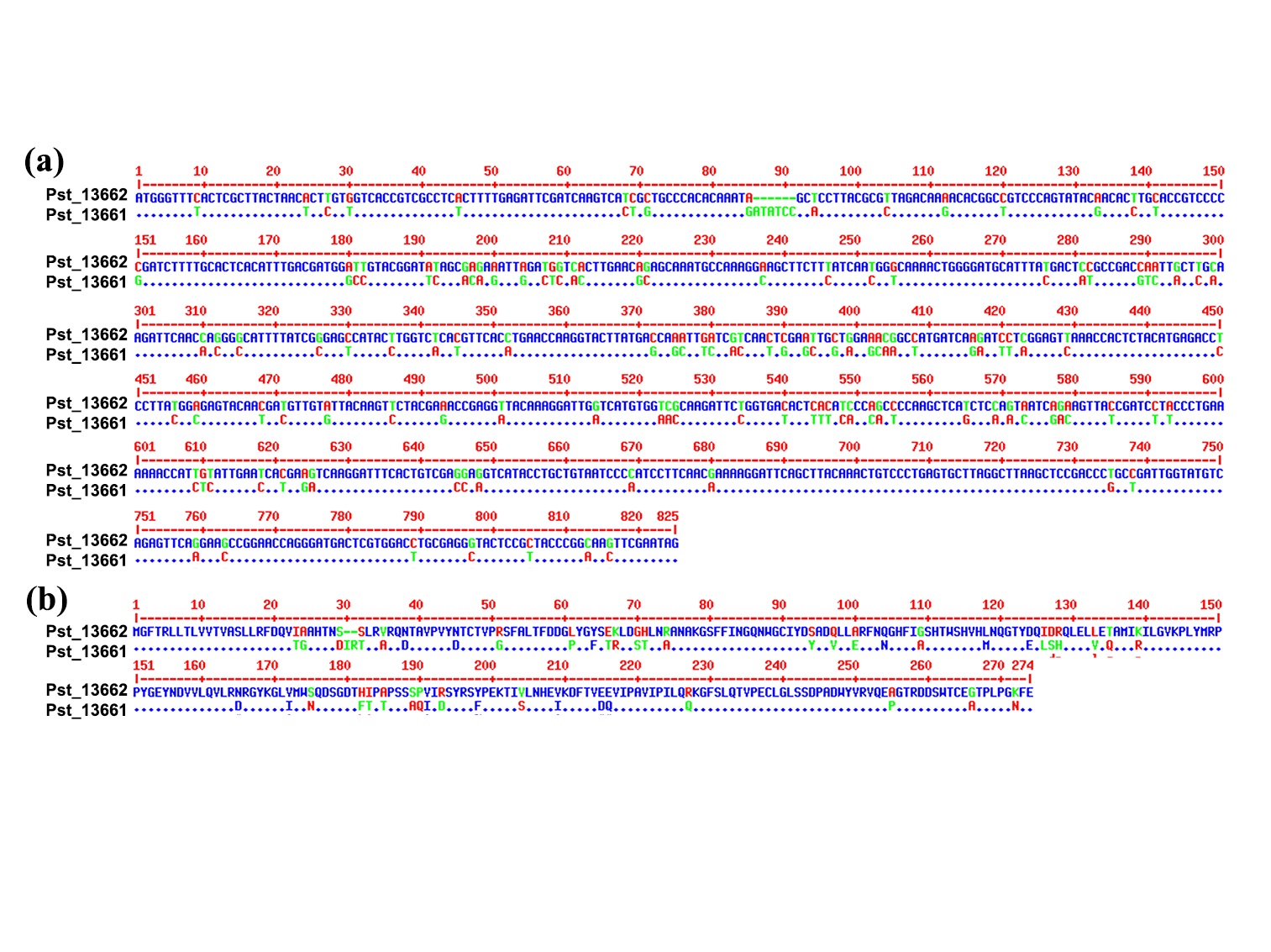

Supplement: Supplementary file 2 — Figure S2. Pst_13661 and Pst_13662 are of high similarity. [file PBI-18-1830-s009.tif]

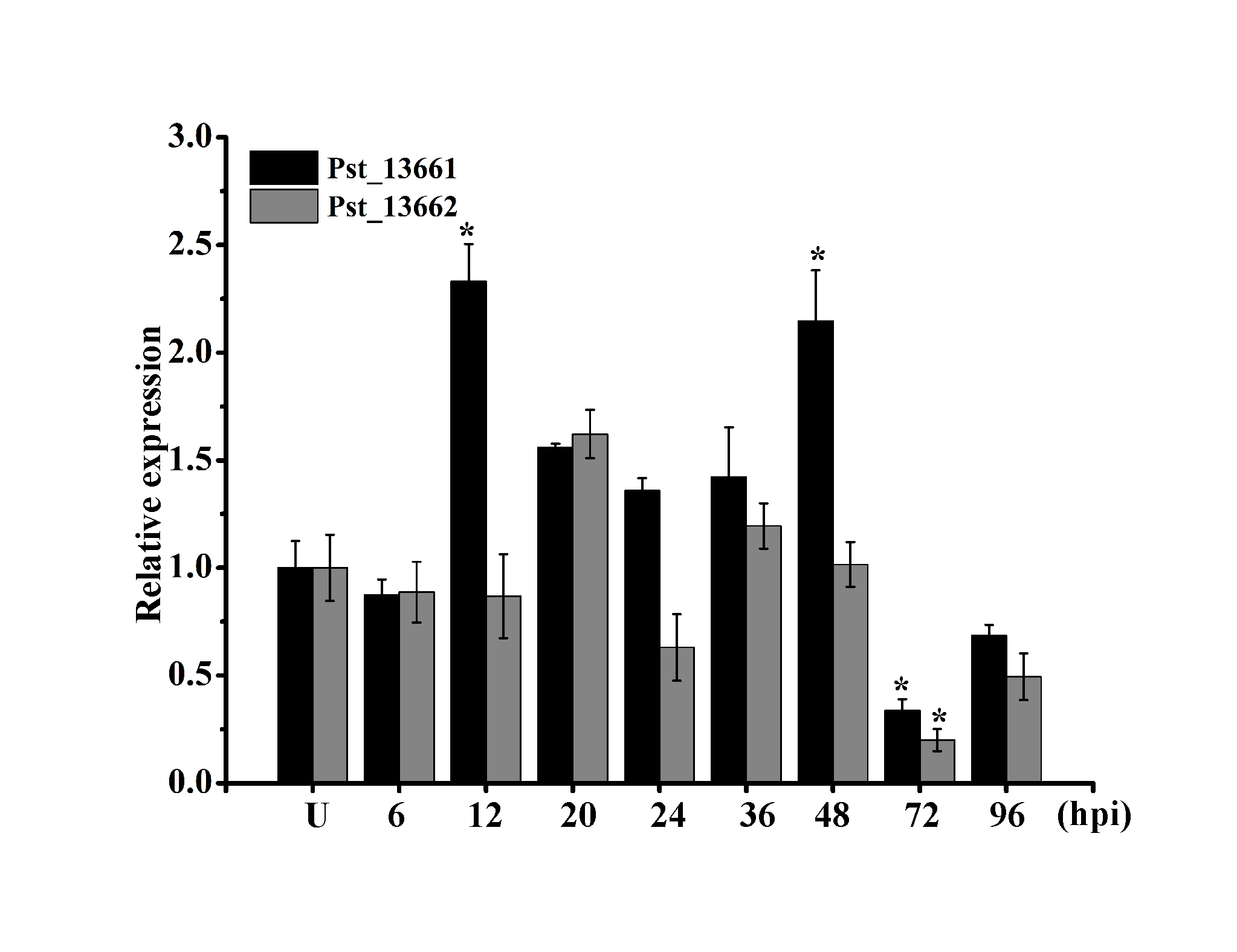

Supplement: Supplementary file 3 — Figure S3. Expression patterns of Pst_13661 and Pst_13662 at different stages infection. [file PBI-18-1830-s008.tif]

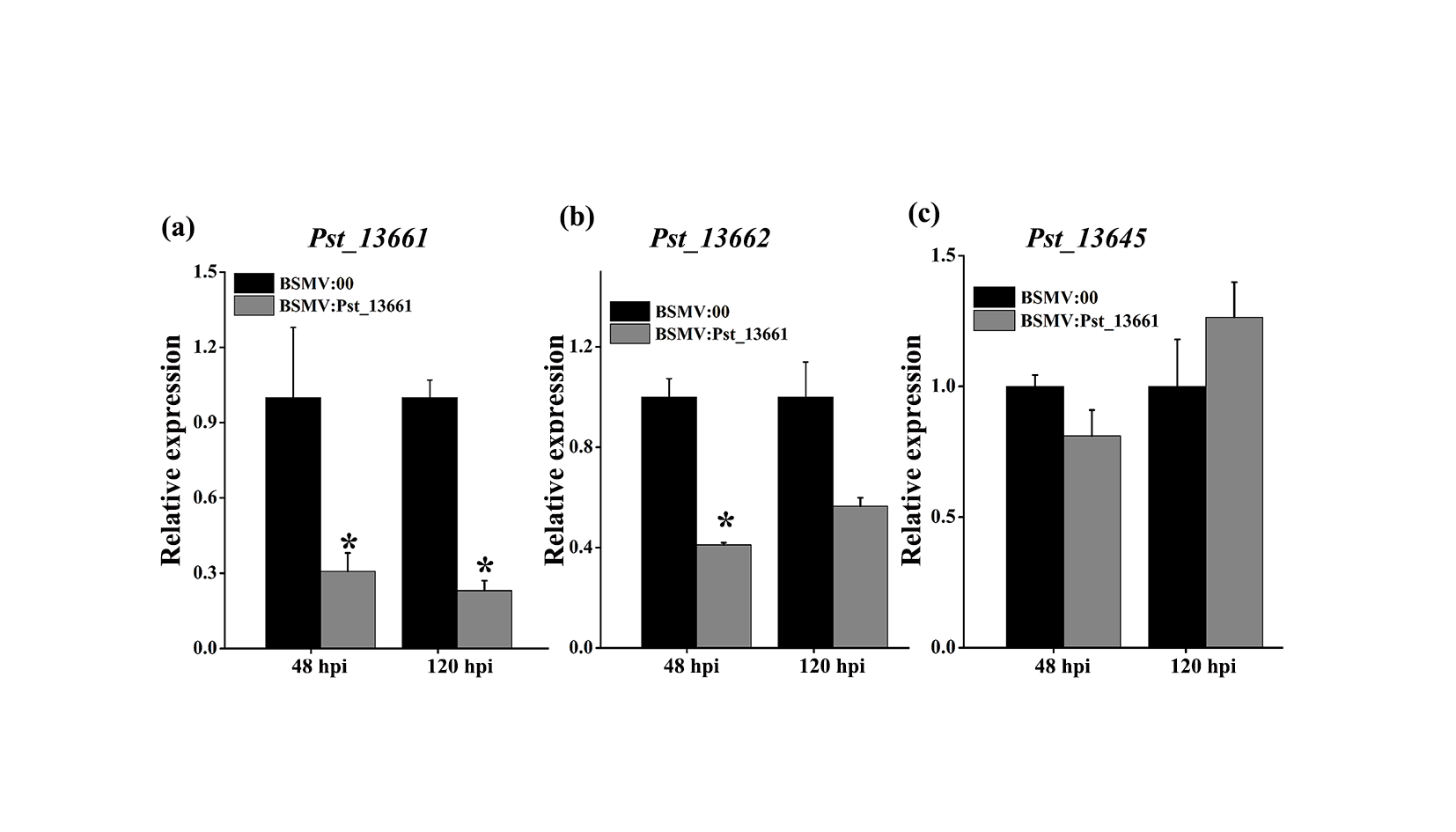

Supplement: Supplementary file 4 — Figure S4. The transcript levels of Pst_13661, Pst_13662 and Pst_13645 were measured in the silenced plants at 48 hpi and 120 hpi. [file PBI-18-1830-s010.tif]

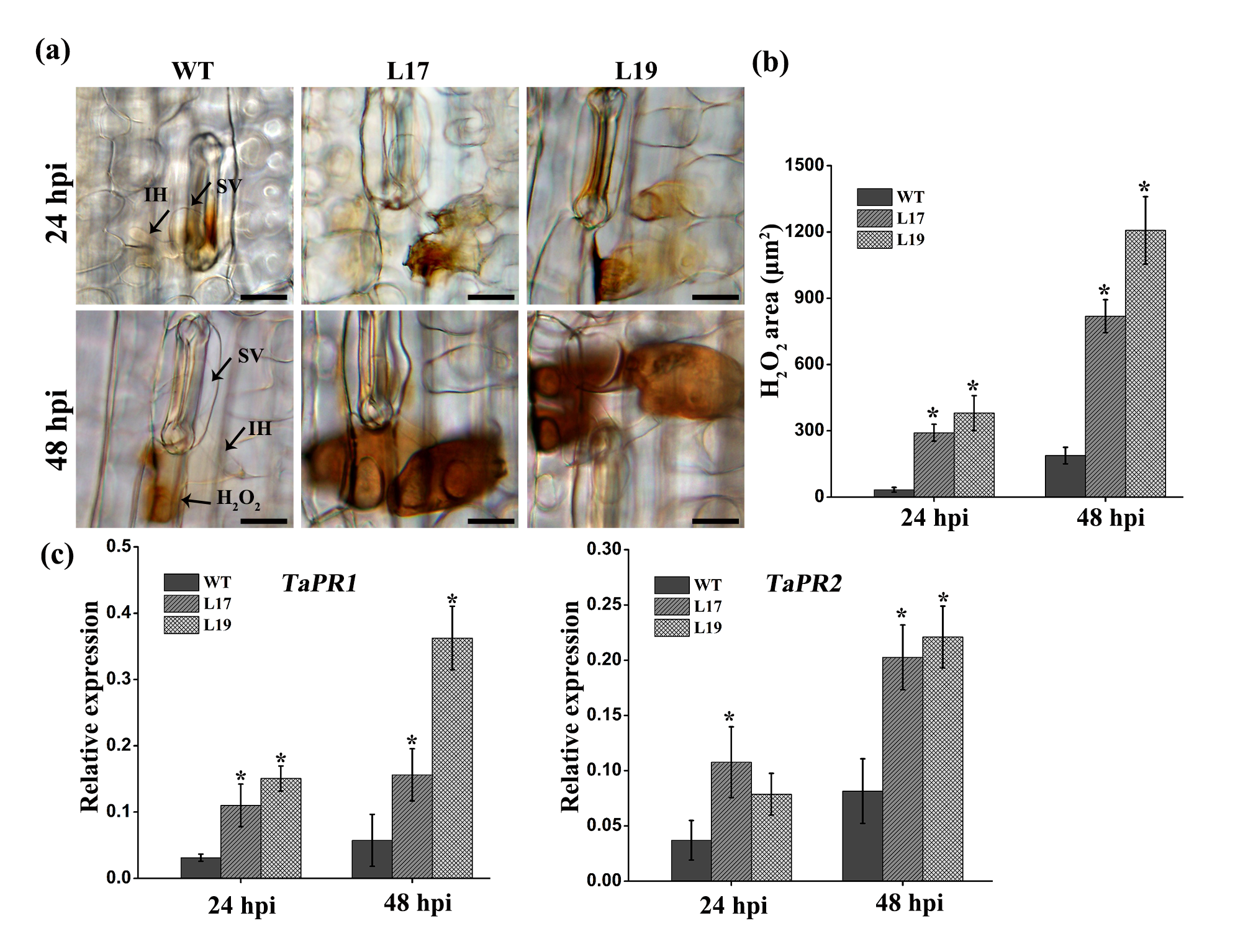

Supplement: Supplementary file 5 — Figure S5. Detection of plant defense response in transgenic plant expressing siRNAs of Pst_13661. [file PBI-18-1830-s001.tif]

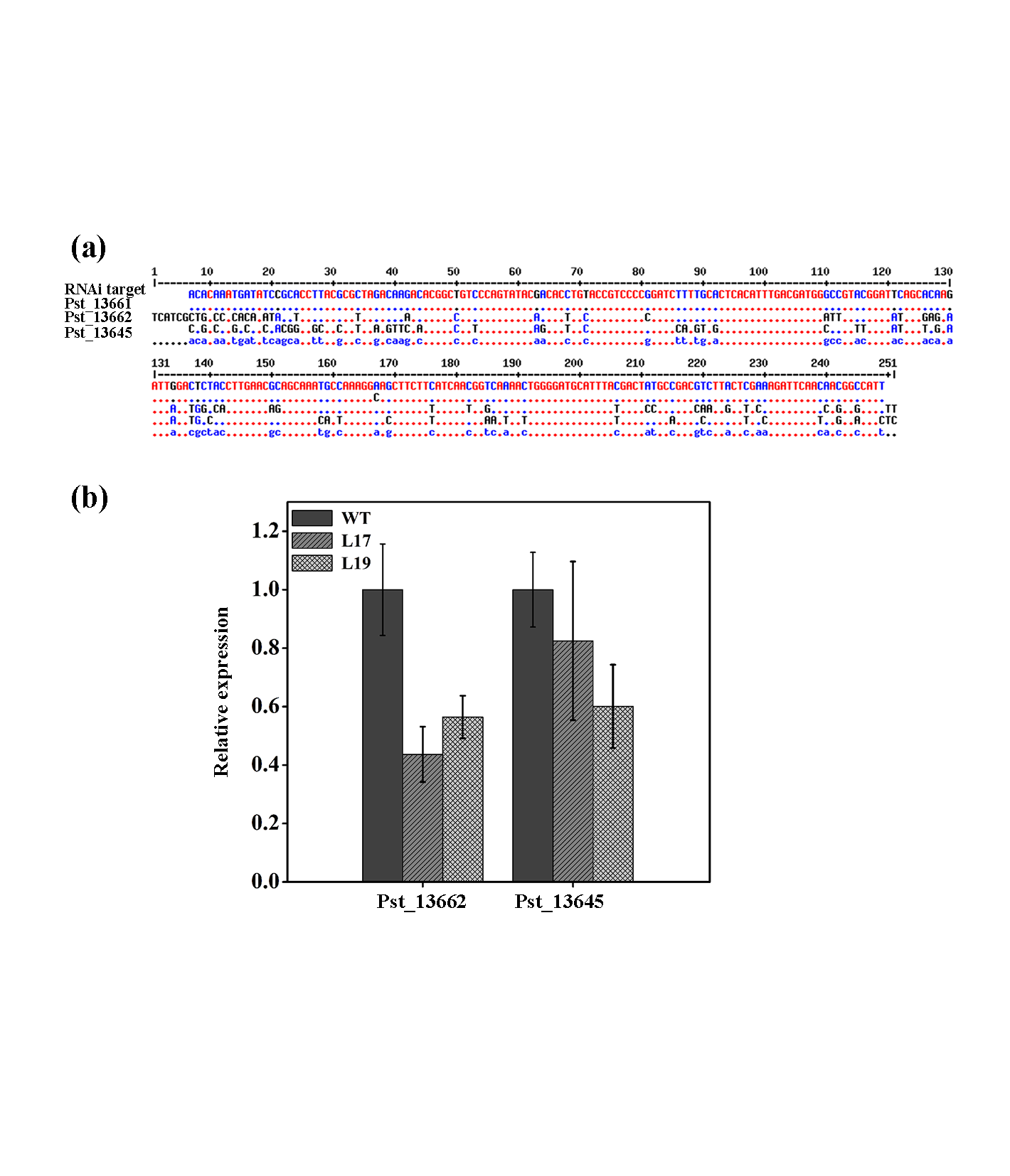

Supplement: Supplementary file 6 — Figure S6. Target of Pst_13661 RNAi transgenic plants and calculation of the silencing efficiency. [file PBI-18-1830-s002.tif]

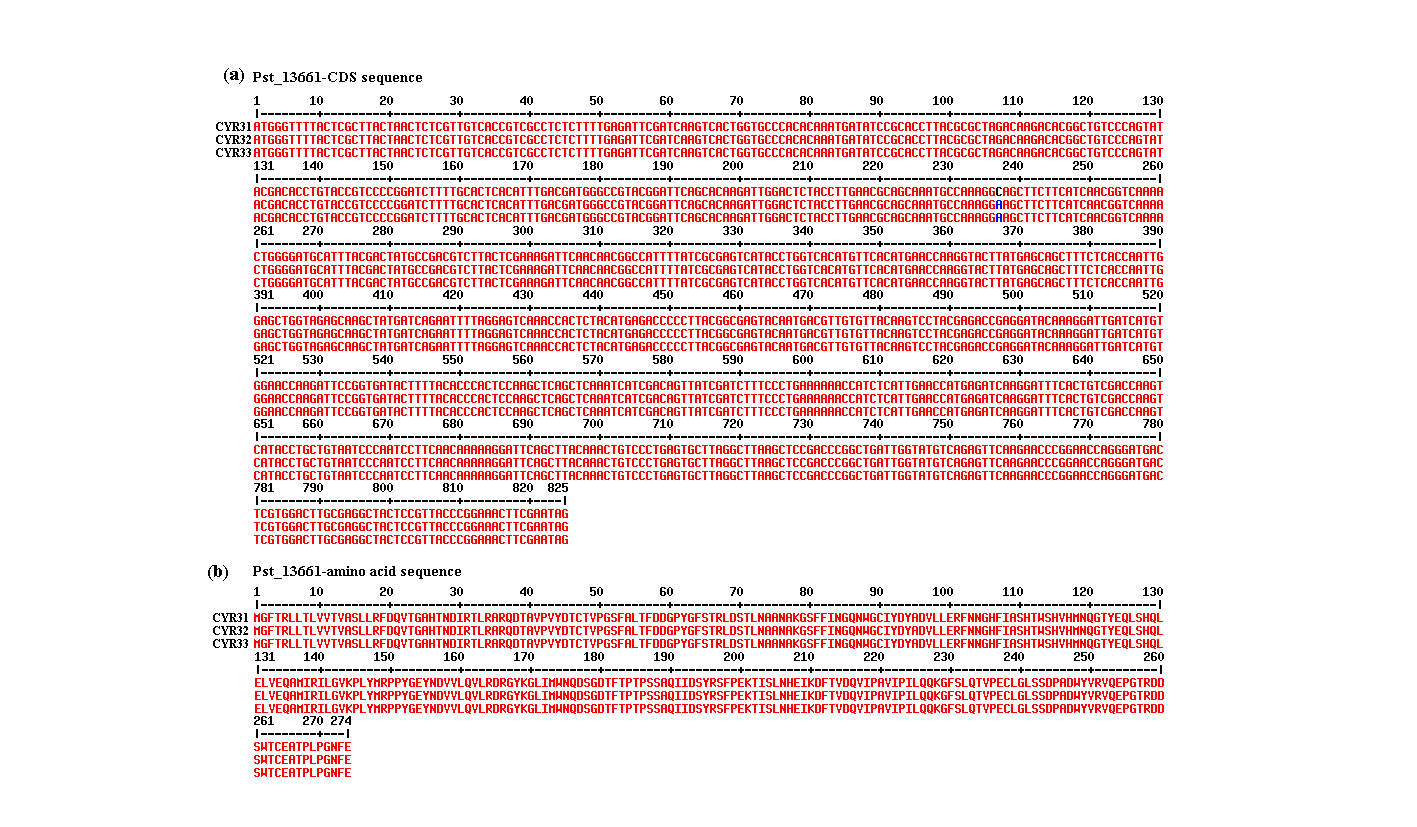

Supplement: Supplementary file 7 — Figure S7. Pst_13661 are of high similarity in main epidemic Pst races. [file PBI-18-1830-s003.tif]

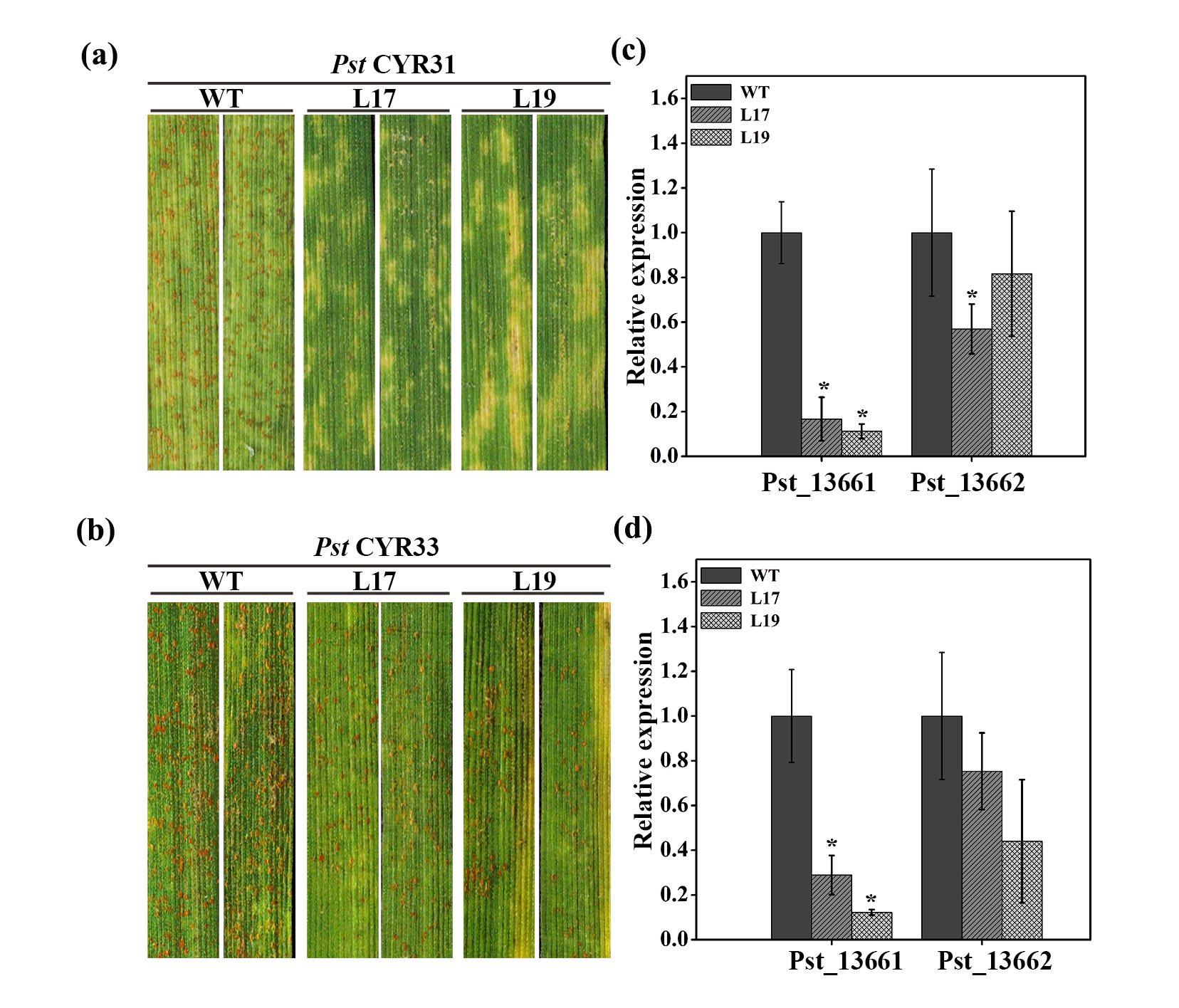

Supplement: Supplementary file 8 — Figure S8. Pathogenicity of main epidemic Pst races CYR31 and CYR33, is impaired in Pst_13661 RNAi transgenic wheat. [file PBI-18-1830-s004.tif]

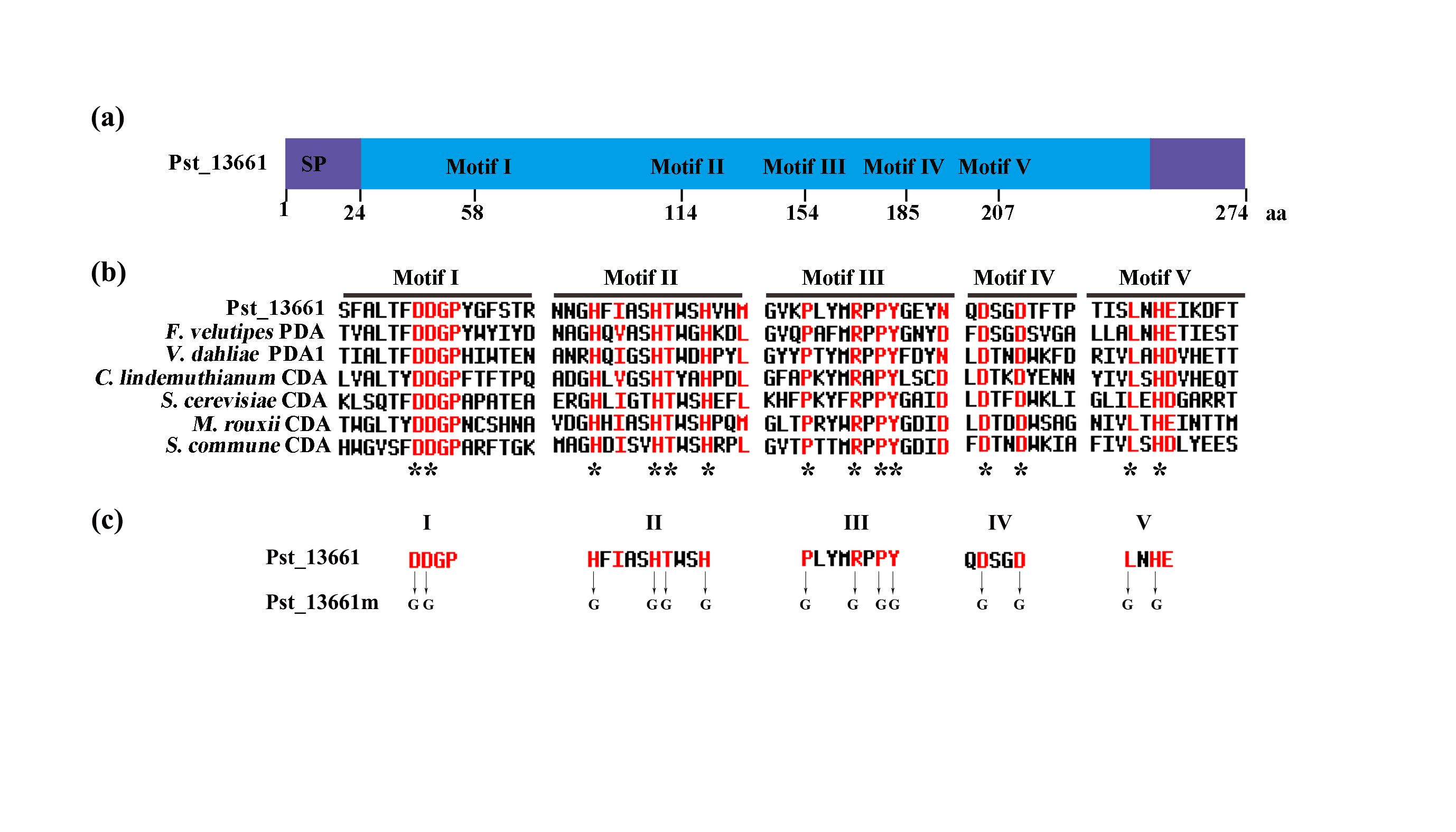

Supplement: Supplementary file 9 — Figure S9. Conserved PDA motifs in Pst_13661. [file PBI-18-1830-s011.tif]
