# Supplementary material for: A polysaccharide deacetylase from Puccinia striiformis f. sp. tritici is an important pathogenicity gene that suppresses plant immunity
Source: Plant Biotechnol J. 2020 Mar 4;18(8):1830–42. doi: 10.1111/pbi.13345 (PMC7336287; doi:10.1111/pbi.13345)
Supplement: Supplementary file 10 — Table S1. Characteristics of five pairs of highly similar gene clusters in Pst genome. [file PBI-18-1830-s006.pdf]

Table S1 Characteristic of 5 pairs of highly similar gene clusters in *Pst* genome

Species	Gene ID	Transcript ID	Similarity (nucleotide)	Similarity (AA)	Signal peptiides (aa)	Structure	Sublocation
Puccinia striiformis f. sp. *tritici PST-78* (ASM14938v1)	PSTG_13661	KNE92947			1-24		apoplastic
	PSTG_13662	KNE92948	80.03%	82.12%	1-24		non-apoplastic, mitochondrial
	PSTG_13644	KNE92928			1-35		apoplastic
	PSTG_13645	KNE92929	58.14%	50.57%	1-32		apoplastic
	PSTG_06370	KNF00442			NO	transmembrane region	
	PSTG_06371	KNF00443	57.86%	47.34%	1-27		Non-apoplastic
	PSTG_06025	KNF00609			1-21		Non-apoplastic
	PSTG_06026	KNF00610	66.91%	63.06%	1-21		Non-apoplastic
	PSTG_03021	KNF03934			NO	transmembrane region	
	PSTG_03021	KNF03935	72.76%	72.77%	NO	transmembrane region	
