# Supplementary material for: A polysaccharide deacetylase from Puccinia striiformis f. sp. tritici is an important pathogenicity gene that suppresses plant immunity
Source: Plant Biotechnol J. 2020 Mar 4;18(8):1830–42. doi: 10.1111/pbi.13345 (PMC7336287; doi:10.1111/pbi.13345)
Supplement: Supplementary file 11 — Table S2. Primers used in this study. [file PBI-18-1830-s007.pdf]

Primer name	Sequence (5'-3')	Application
Pst_13661-F	ggaattcATGGGTTTTACTCGCTTACTAAC	
Pst_13661-R	ccgctcgagACCAGTGACTTGATCGAATCTC	Secretion assay
Pst_13661-F	ggcatcgatTACCCATACGACGTCCCAGACTACGCTATGGGTTTTACTCGCTTACTAAC	
Pst_13661-R	catgtcgacCTATTCGAAGTTTCCGGGTAAC	Suppression Bax-PCD
Pst_13661-F	gGAATTCATGGCCCACACAAATGATATC	
Pst_13661-R	cgGGATCCCTATTCGAAGTTTCCGGGTAAC	
Pst_13661-HA-F	TTACAATTATCGATACAATGTACCCATACGACGTCCCAGACTACGCTATGGCCCACACAAATGATATC	
Pst_13661-HA-R	CTCATTAAAGCAGGACAAGCCTATTCGAAGTTTCCGGGTAAC	
Pst_13661-Flag-F	TTACAATTATCGATACAATGGATTACAAGGATGACGACGATAAGATGGCCCACACAAATGATATC	
Pst_13661-F	cgGGATCCATGGGTTTTACTCGCTTACTAAC	
Pst_13661-R	cctcgagTTCGAAGTTTCCGGGTAAC	Interaction assay
Pst_13662DNA-F	CCTTGCCAGCTCACCCTTC	
Pst_13662DNA-R	AGACAAATCGCCTCAAAGTCCT	
Pst_13661DNA-F	ACACTCTCCCAAGGAAATCATC	
Pst_13661DNA-R	GGAGCTCAACAAGGTATGAAAG	Amplification gene
PstEF-F	TTCGCCGTCCGTGATATGAGACAA	
PstEF-R	ATGCGTATCATGGTGGTGGAGTGA	
TaEF-F	TGGTGTCATCAAGCCTGGTATGGT	
TaEF-R	ACTCATGGTGCATCTCAACGGACT	
1Pst_3661-qrt-F	CTCGTTGTCACCGTCGC	
Pst_13661-qrt-R	AGGTAGAGTCCAATCTTGTGCT	
Pst_13662-qrt-F	CATCCCAGCCCCAAGC	
Pst_13662-qrt-R	GCAGGTCCACGAGTCATCC	
Pst_13645-qrt-F	GGAATGGGGTTTGCTC	
Pst_13645-qrt-R	CTTTAGTGTTTGCTGCGTT	
NbAct-F	GTTGCTATACAAGCTGTTCTCTCG	
NbAct-R	GTCAAGACGAAGAATGACATGTGG	
NbPR1a-F	CGACCAGGTAGCAGCCTATG	
NbPR1a-R	TCTCAACAGCCTTAGCAGCC	
NbPR2-F	GGGCTGTTAATTTGCAGTATCC	
NbPR2-R	GGTTTATAACATCTTGGTCTGATGG	
NbWRKY12-F	CTCATCAGCTAGTTCATTTGATGC	
NbWRKY12-R	AGCTCGGTCTTTGTTCTAAAAGC	
TaPR1-F	GAGAATGCAGACGCCCAAGC	
TaPR1-R	CTGGAGCTTGCAGTCGTTGATC	
TaPR2-F	AGGATGTTGCTTCCATGTTTGCCG	
TaPR2-R	AAGTAGATGCGCATGCCGTTGATG	qRT-PCR
Pst_13661-HIGS-F	ataTTAATTAAGCAAATGCCAAAGGAAGCTT	
Pst_13661-HIGS-R	tatGCGGCCGCCAATCCTTTGTATCCTCGGTCT	HIGS assay
13661-mCherry-F	TTACAATTATCGATACAATGATGGGTTTTACTCGCTTACTAAC	
13661-mCherry-R	CACCATCCTAGGACTAGTTTGAGCGTAGTCTGGGACGTCGTATGGGTATTCGAAGTTTCCGGGTAAC	Sub-localizaiton
13661-RNAi-S	ACACAAATGATATCCGCACCTT	Identification of transgenetic plant
13661-RNAi-AS	AATGGCCGTTGTTGAATCTTT	
